# Supplementary material for: Selection Signatures in Worldwide Sheep Populations
Source: PLoS One. 2014 Aug 15;9(8):e103813. doi: 10.1371/journal.pone.0103813 (PMC4134316; doi:10.1371/journal.pone.0103813)
Supplement: File S1 — Combined Supporting Information File including Figures S1 to S9, Tables S1 to S3 and Text S1. (PDF) [file pone.0103813.s003.pdf]

Selection signatures in worldwide Sheep populations - Supplementary material

Maria-Ines Fariello[1], Bertrand Servin[1], Gwenola Tosser-Klopp[1], Rachel Rupp[1], Carole Moreno[1], Magali SanCristobal[1], Simon Boitard[3,4,*]

1 Génétique, Physiologie et Systèmes d'Élevage, INRA & ENVT & ENSAT, Castanet-Tolosan, France

3 Génétique Animale et Biologie Intégrative, INRA & AgroParisTech, Jouy-en-Josas, France

4 Origine, Structure et Evolution de la Biodiversité, Museum National d'Histoire Naturelle & EPHE & CNRS, Paris, France

∗ **E-mail: Corresponding sboitard@mnhn.fr**

Candidate Gene	Populations
BNC2	COM
KITLG	COM, EMZ
KIT	VBS
EDN3	MOG
MC1R	SUF, SAB, GAL
ASIP	MER
MITF	VBS, ERS, BOS

Table S1. List of genes potentially associated with coloration patterns found under selection signatures and the most likely candidate populations for selection.

group	K	μ	σ
AFR	5	1.97	0.53
ASI	25	7.78	0.92
CEU	10	3.41	0.62
ITA	15	2.83	0.44
NEU	40	4.09	0.45
SWA	10	3.28	0.55
SWE	25	3.32	0.39

Table S2. Parameters for the hapFLK genome scan. K : Number of haplotype clusters used in the LD model for each group, as determined by the fastPHASE cross-validation procedure. μ,σ mean and standard deviation of the normal distribution used to model the hapFLK neutral distribution

group	DF	$\overline{FLK}$
AFR	1	0.97
ASI	7	6.65
CEU	3	2.91
ITA	3	2.93
NEU	5	4.78
SWA	3	2.92
SWE	3	2.96

Table S3. Theoretical number of degrees of freedom (DF) and observed mean of the FLK statistic ($\overline{FLK}$) in each group of populations.

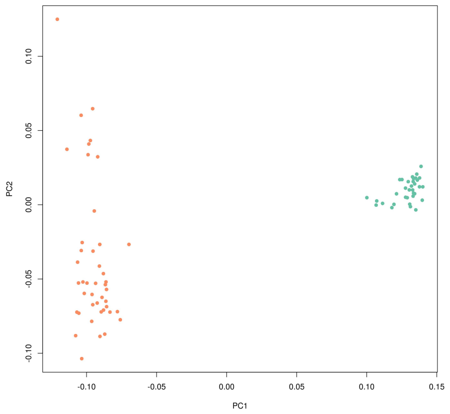

Figure S1. Projection of animals from the African group on the first 2 principal components.

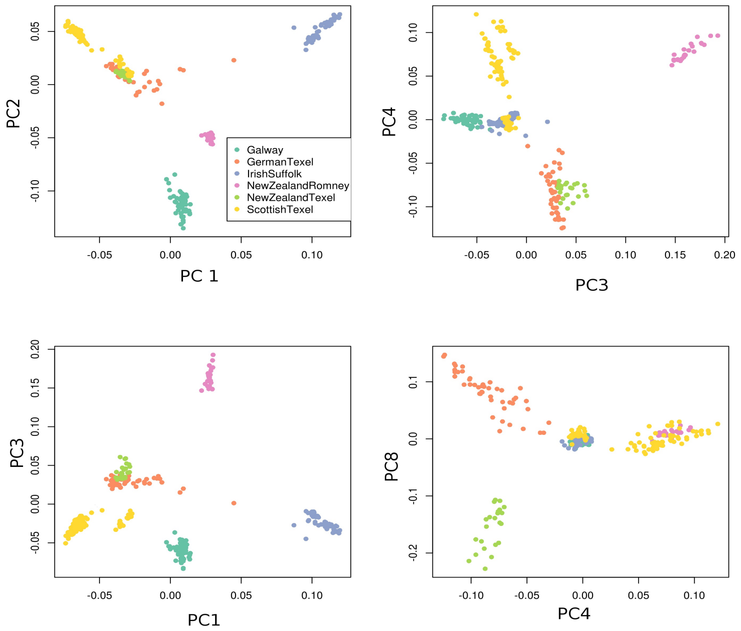

Figure S2. Projection of animals from the North European group on the first 8 principal components.

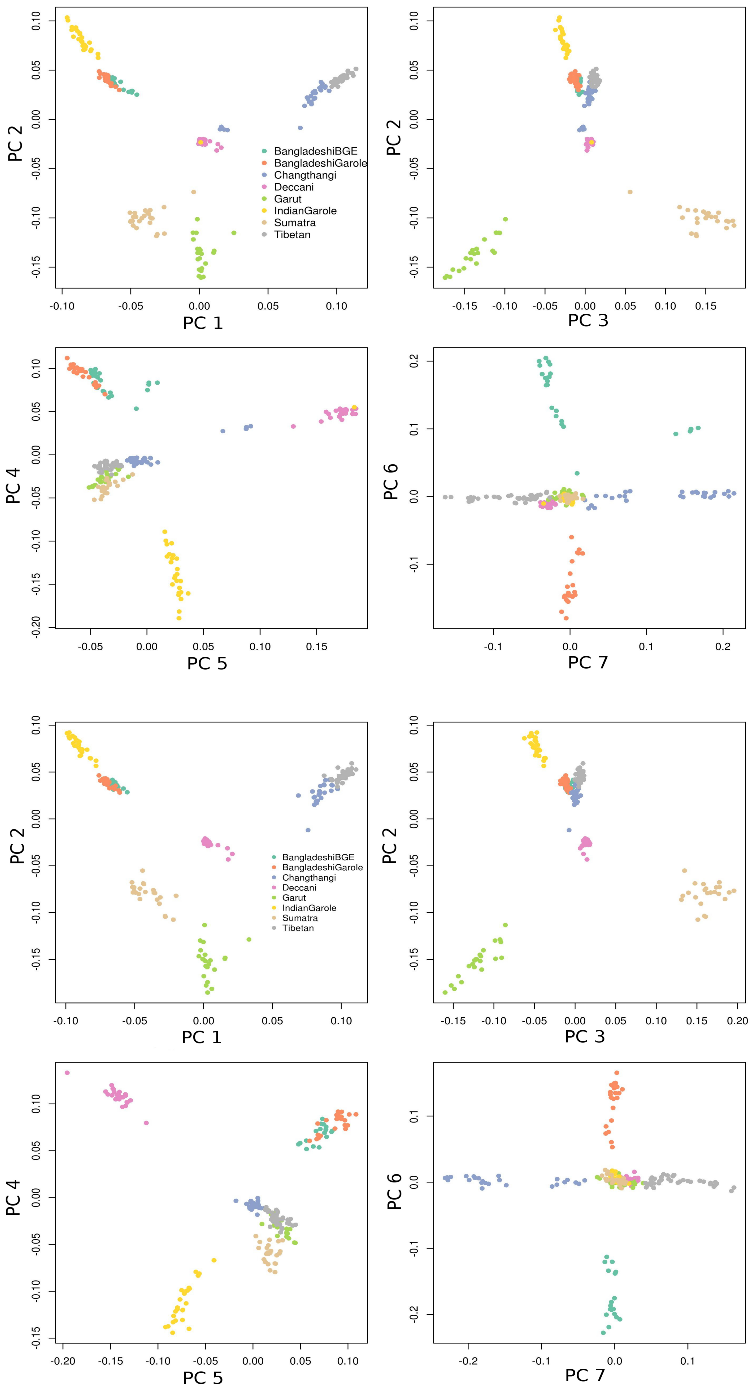

Figure S3. Projection of animals from the Asian group on the first 8 principal components, before (top) and after (bottom) correction. One Indian Garole animal located in the Deccani cluster was attributed to this breed. Five Bangladeshi BGE animals clustering away from the rest of the breed were removed. Four Changthangi animals, which clustered away from the rest of the breed and appeared admixed with the Deccani breed, were removed. One outlier Sumatra animal was also removed.

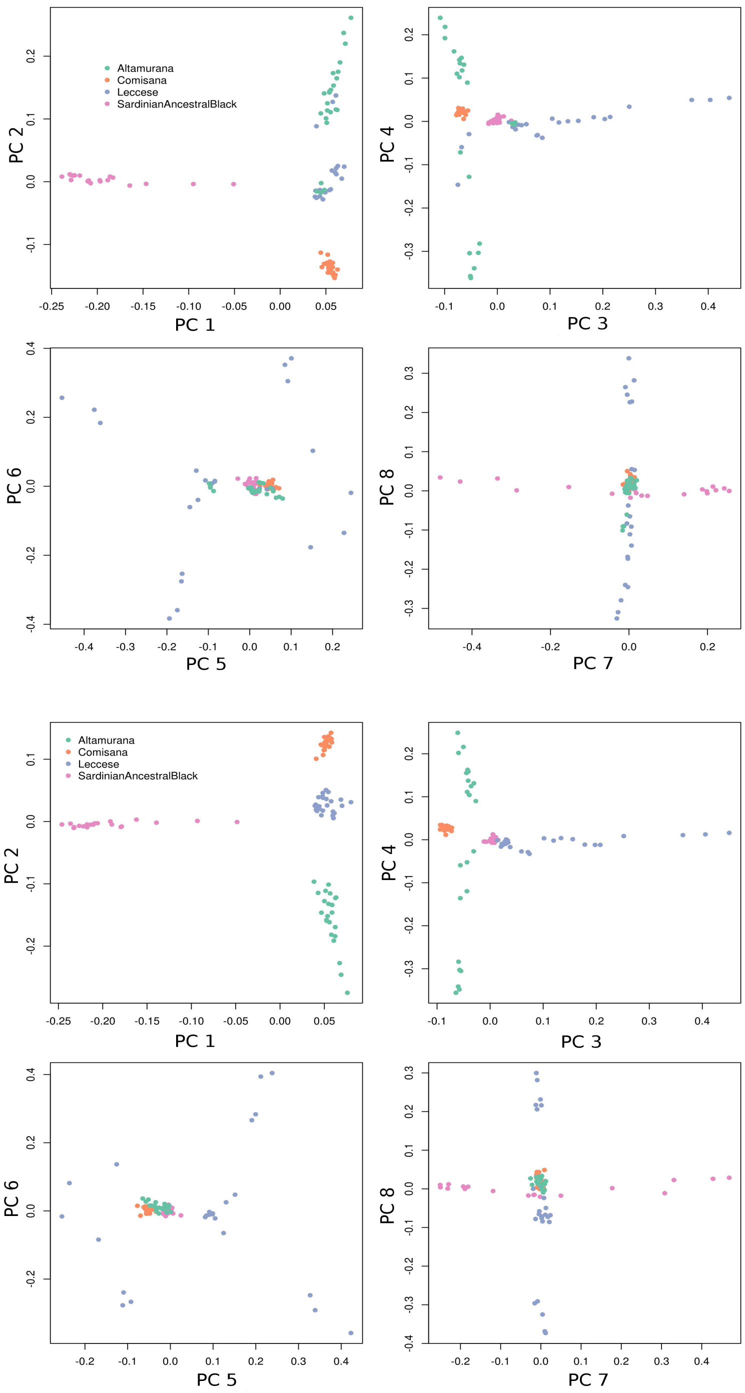

Figure S4. Projection of animals from the Italian group on the first 8 principal components, before (top) and after (bottom) correction. Four Altamurana animals located in the Leccese cluster were attirubted to the Leccese breed. Similarly, three Lecese animals located in the Altamurana cluster was attributed to the Altamurana cluster.

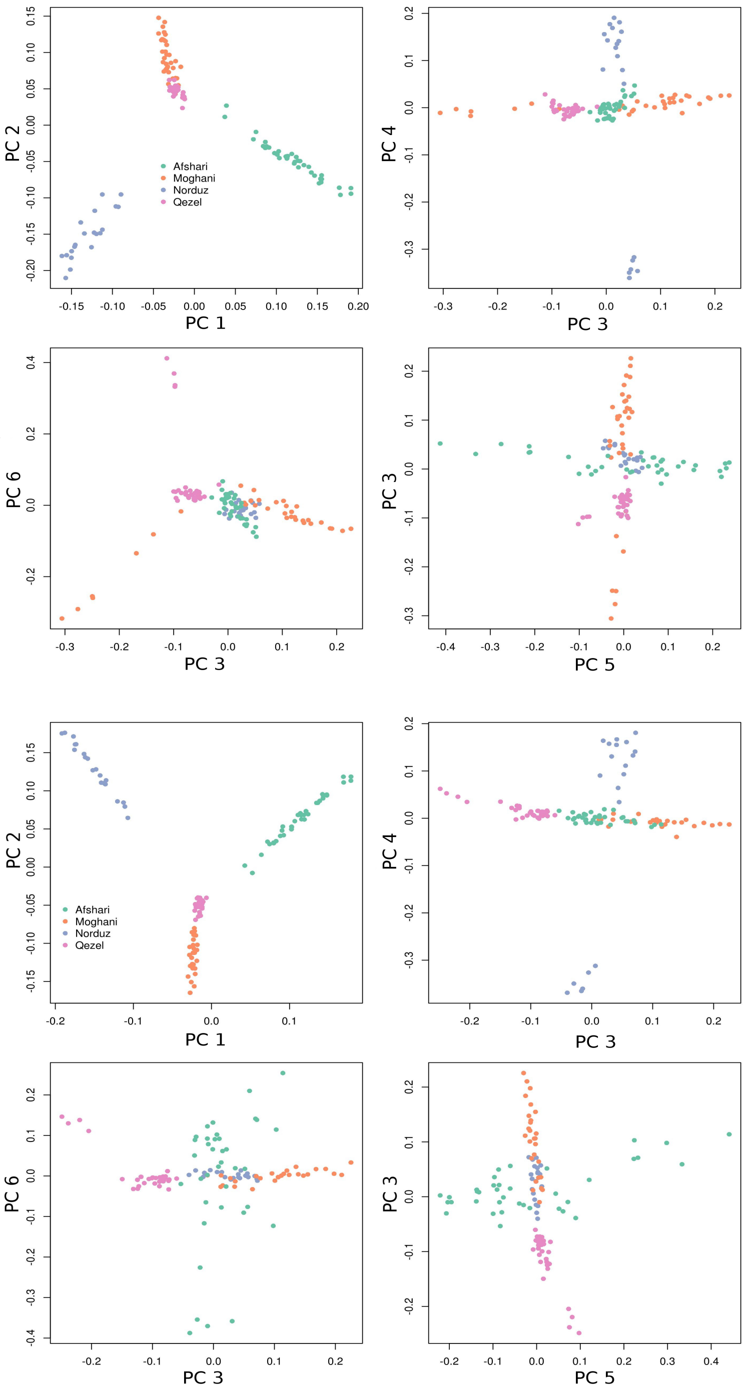

Figure S5. Projection of animals from the South West Asian group on the first 8 principal components, before (top) and after (bottom) correction. Seven Moghani animals clustering away from the rest of the breed, and possibly admixed with the Qezel breed, were removed.

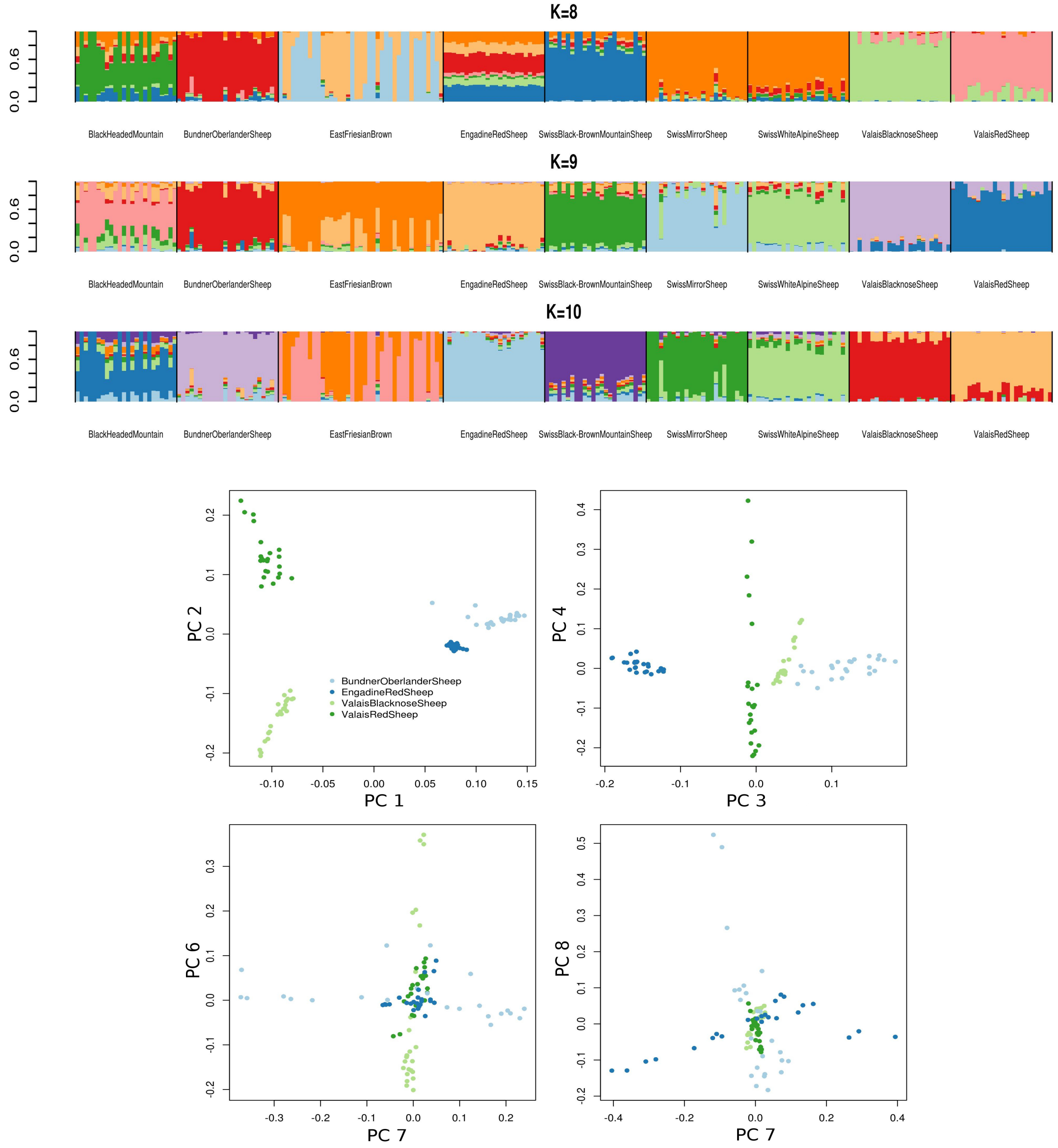

Figure S6. Admixture analysis for animals of the Central European group (top), and projection of the selected animals on the first 8 principal components (bottom). Three breeds (Swiss Mirror Sheep, Swiss Alpine White Sheep and Swiss Black-Brown Mountain Sheep) were reported as admixed breeds in [1] and were consequently removed, although they appeared relatively homogeneous in the admixture analysis. Two further breeds (East Friesian Brown and Black Headed Mountain) were removed based on the admixture analysis.

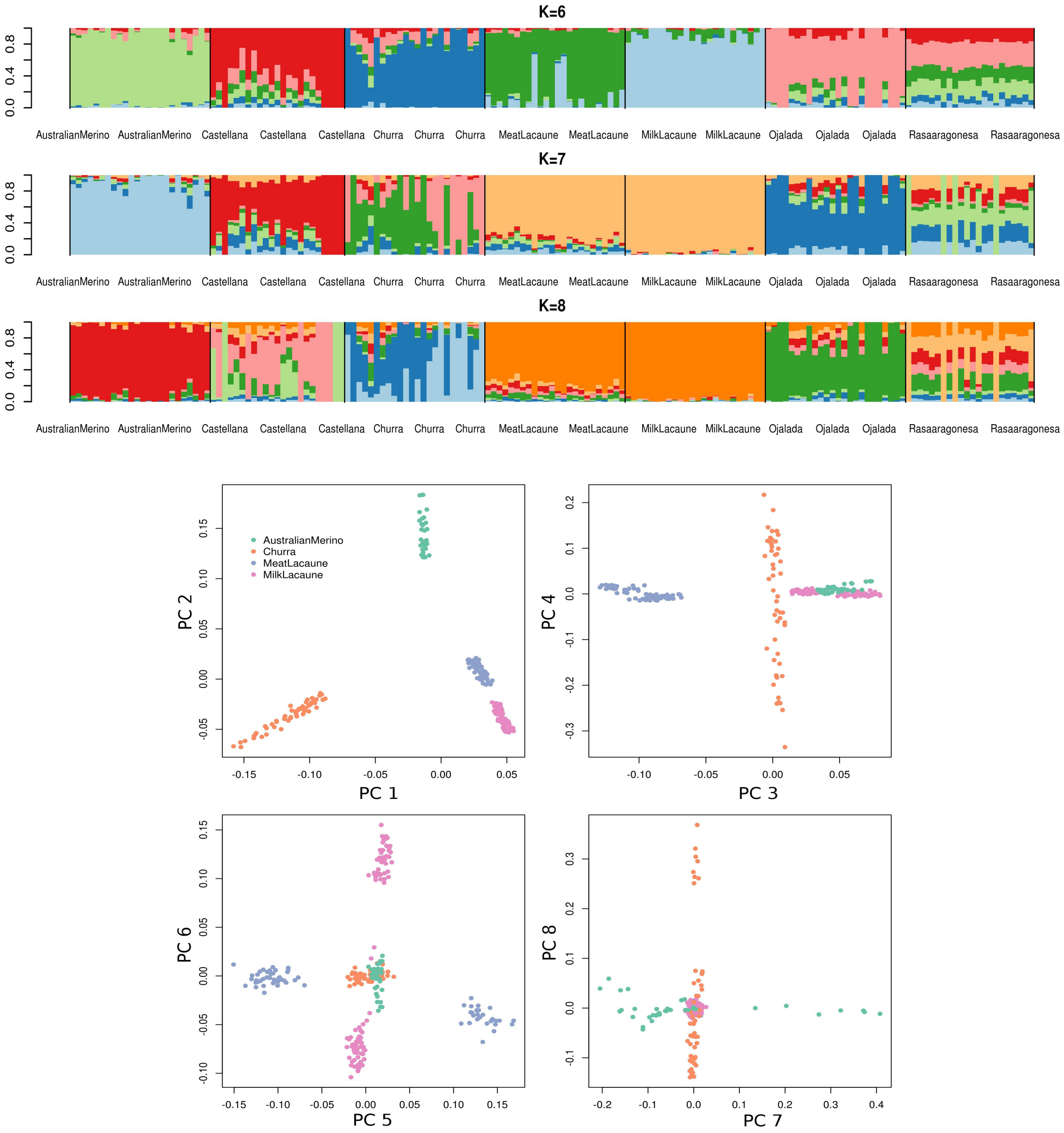

Figure S7. Admixture analysis for animals of the South West European group (top), and projection of the selected animals on the first 8 principal components (bottom). For the admixture analysis, a subset of 24 animals was sampled at random within each breed in order to obtain balanced sample sizes. Three breeds (Castellana, Ojalada and Rasaaragonesa) were removed based on the admixture analysis, as they were clearly admixed. Historical records concerning those 3 breeds are ambiguous, some of them reporting that they may result from a cross between Merino and Churra animals. The Churra breed also looked admixed in this analysis, but this was no longer the case after removing the three other problematic breeds.

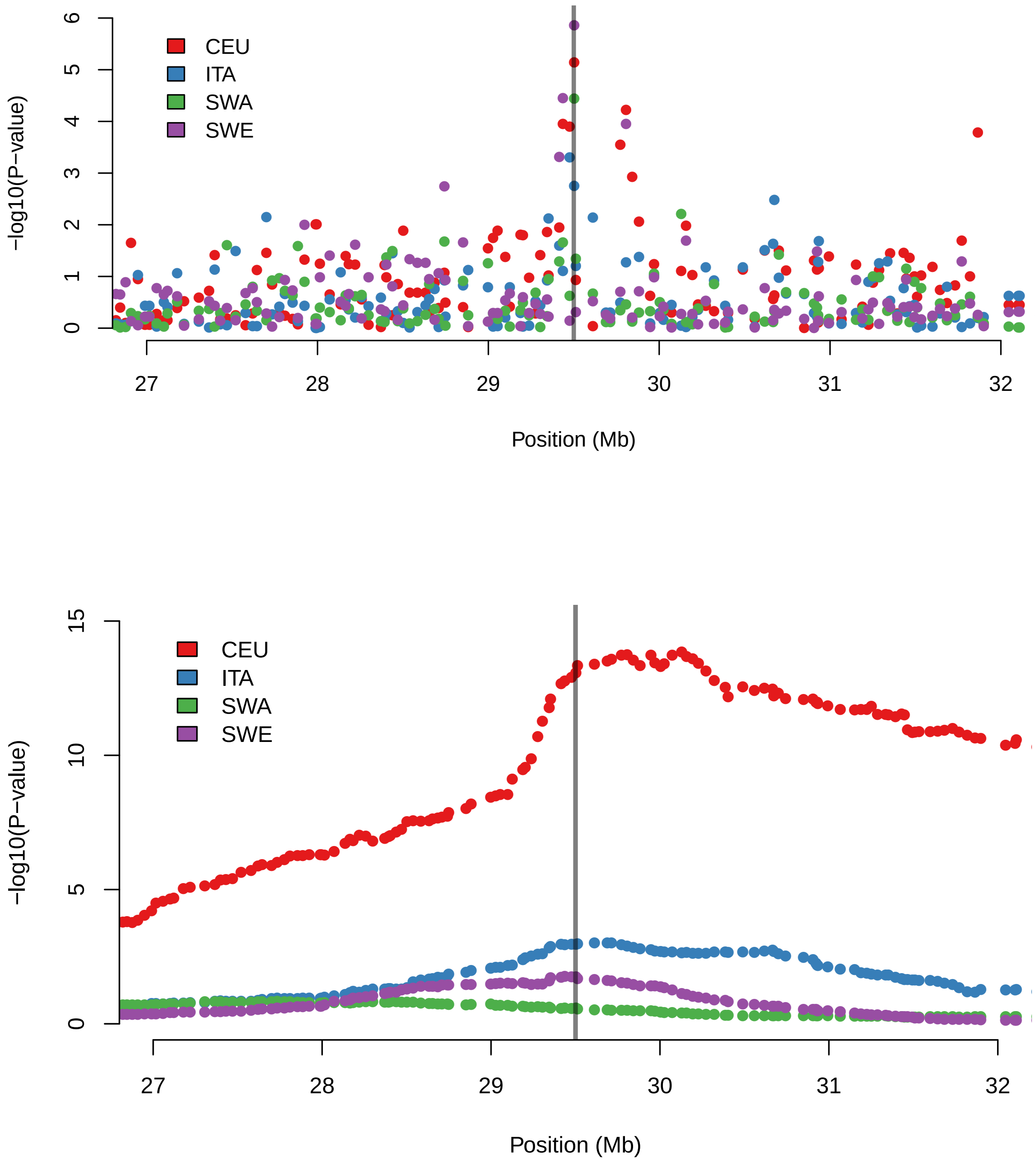

Figure S8. FLK (top) and hapFLK (bottom) p-values in the region of RXFP2. The position of RXFP2 is indicated by the vertical gray line.

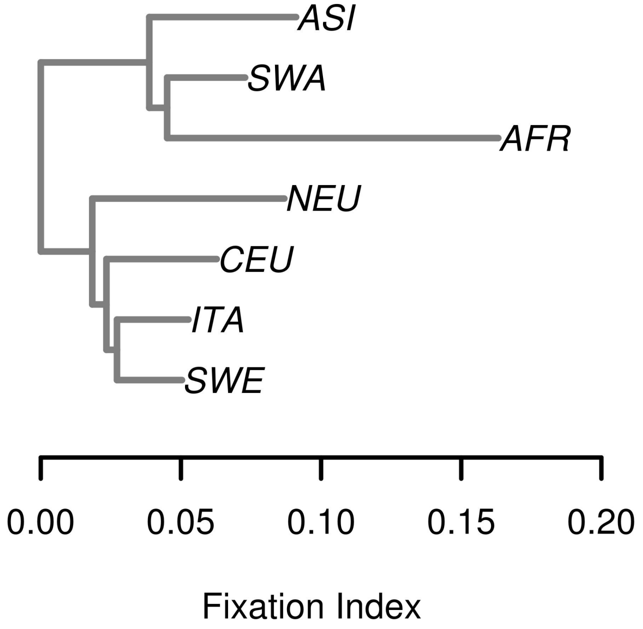

Figure S9. Phylogenetic tree of the ancestral populations of geographical groups.

References

1. Kijas JW, Lenstra JA, Hayes B, Boitard S, Porto Neto LR, et al. (2012) Genome-wide analysis of the world's sheep breeds reveals high levels of historic mixture and strong recent selection. PLoS Biol 10: e1001258.

Text S1. FLK and hapFLK genome scans within groups of populations

For each population group, we show, in this order

1. histogram of the observed FLK distribution, and corresponding theoretical χ^2 distribution
2. the FLK p-value distribution
3. histogram of the hapFLK distribution and corresponding estimated normal distribution (see details in Methods)
4. the hapFLK p-value distribution
5. Manhattan plot of the FLK p-values
6. Manhattan plot of the hapFLK p-values

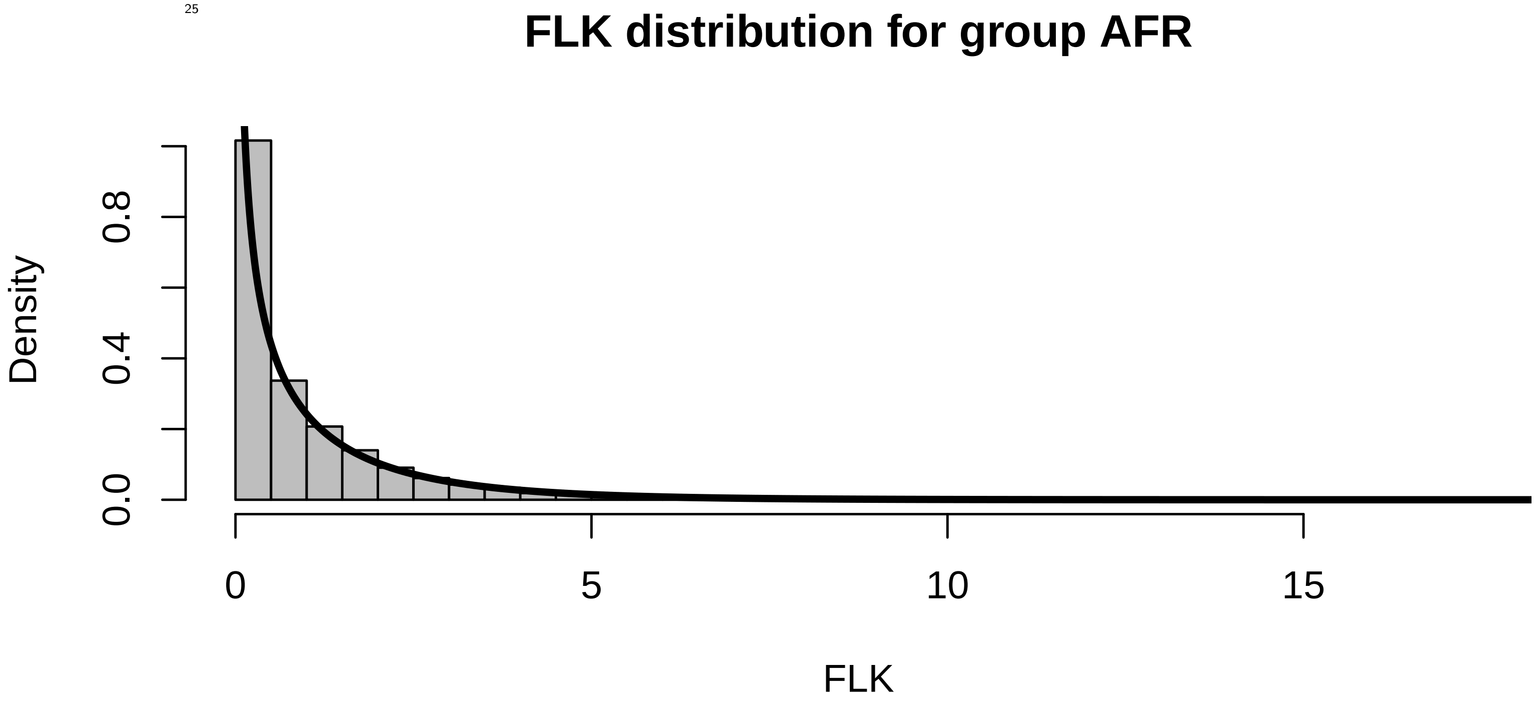
FLK distribution for group AFR
Density
0.0
0.4
0.8
0
5
10
15
FLK

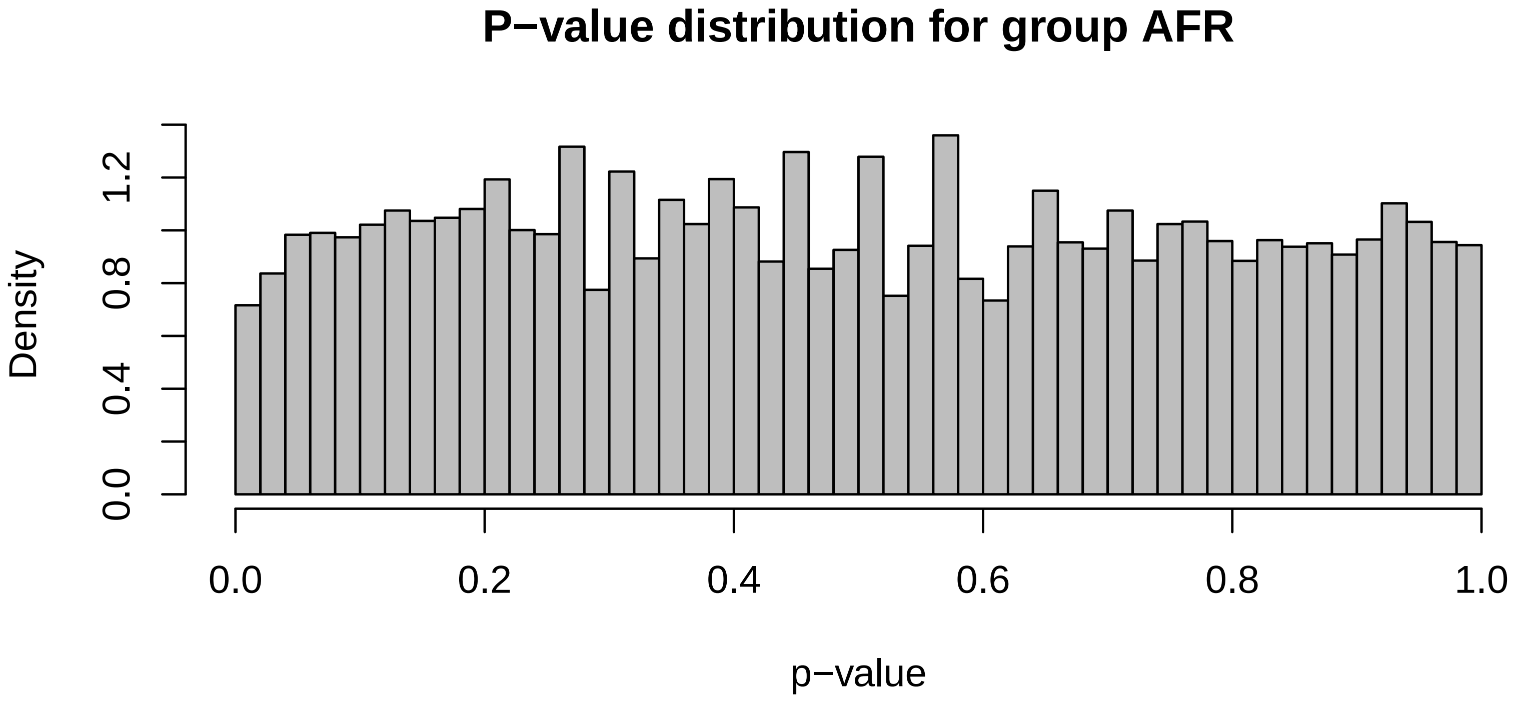
P−value distribution for group AFR
Density
0.0
0.4
0.8
1.2
0.0
0.2
0.4
0.6
0.8
1.0
p−value

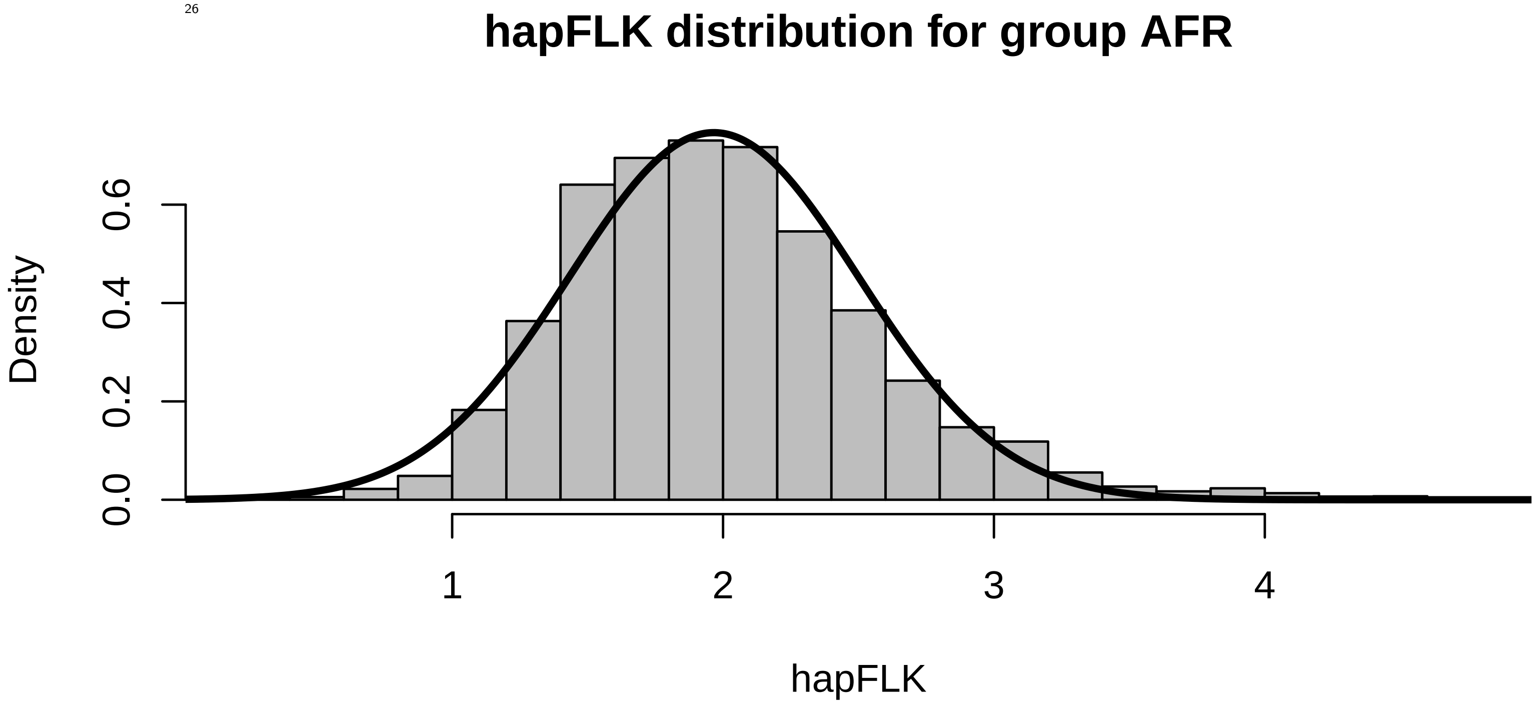
hapFLK distribution for group AFR
Density
0.0
0.2
0.4
0.6
1
2
3
4
hapFLK

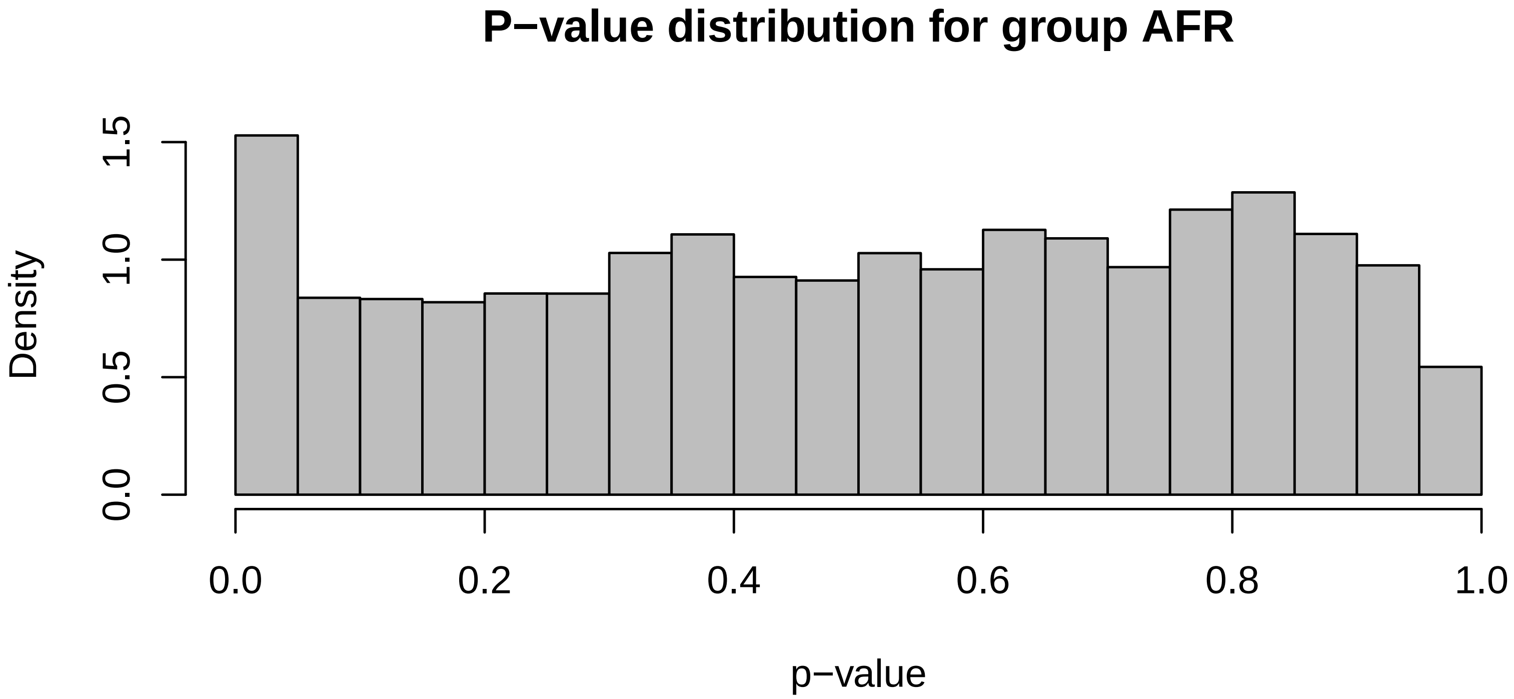
P−value distribution for group AFR
Density
0.0
0.5
1.0
1.5
0.0
0.2
0.4
0.6
0.8
1.0
p−value

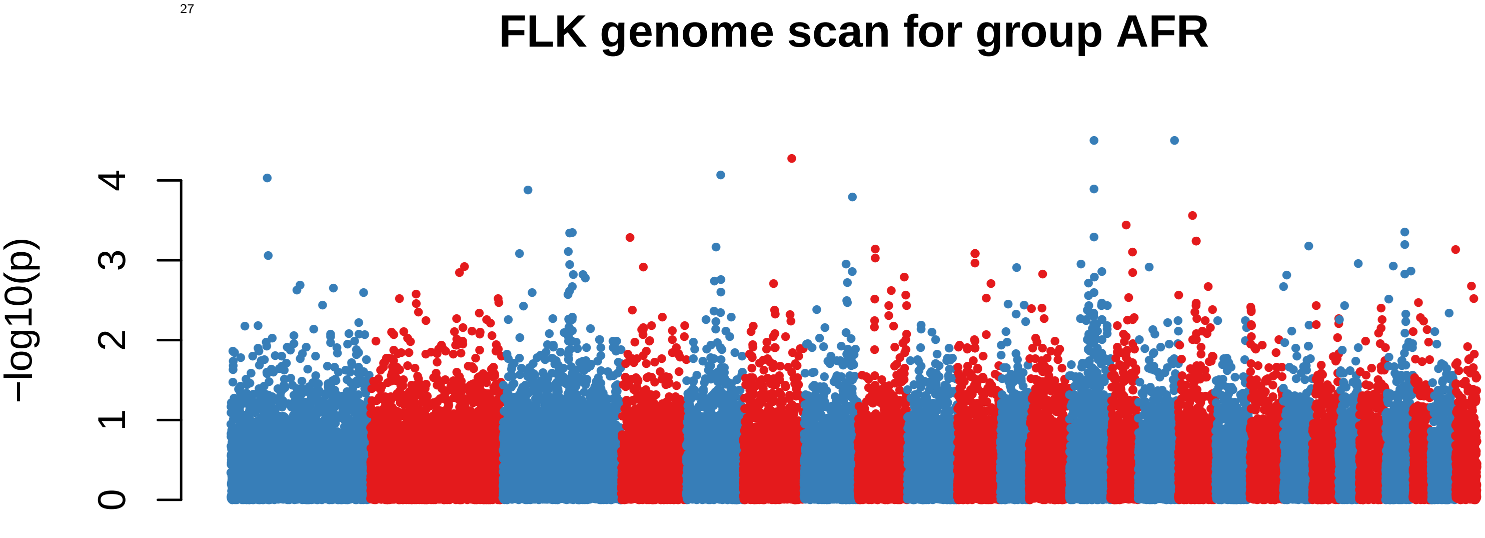
FLK genome scan for group AFR
−log10(p)
0
1
2
3
4

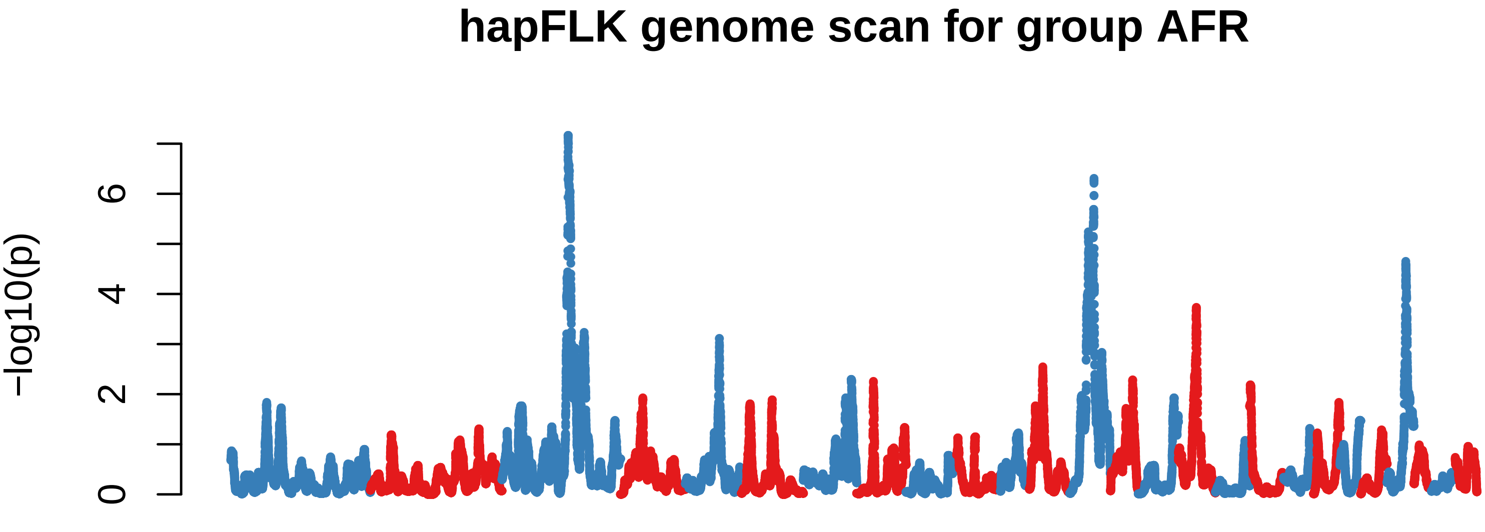
hapFLK genome scan for group AFR
−log10(p)
0
2
4
6

28

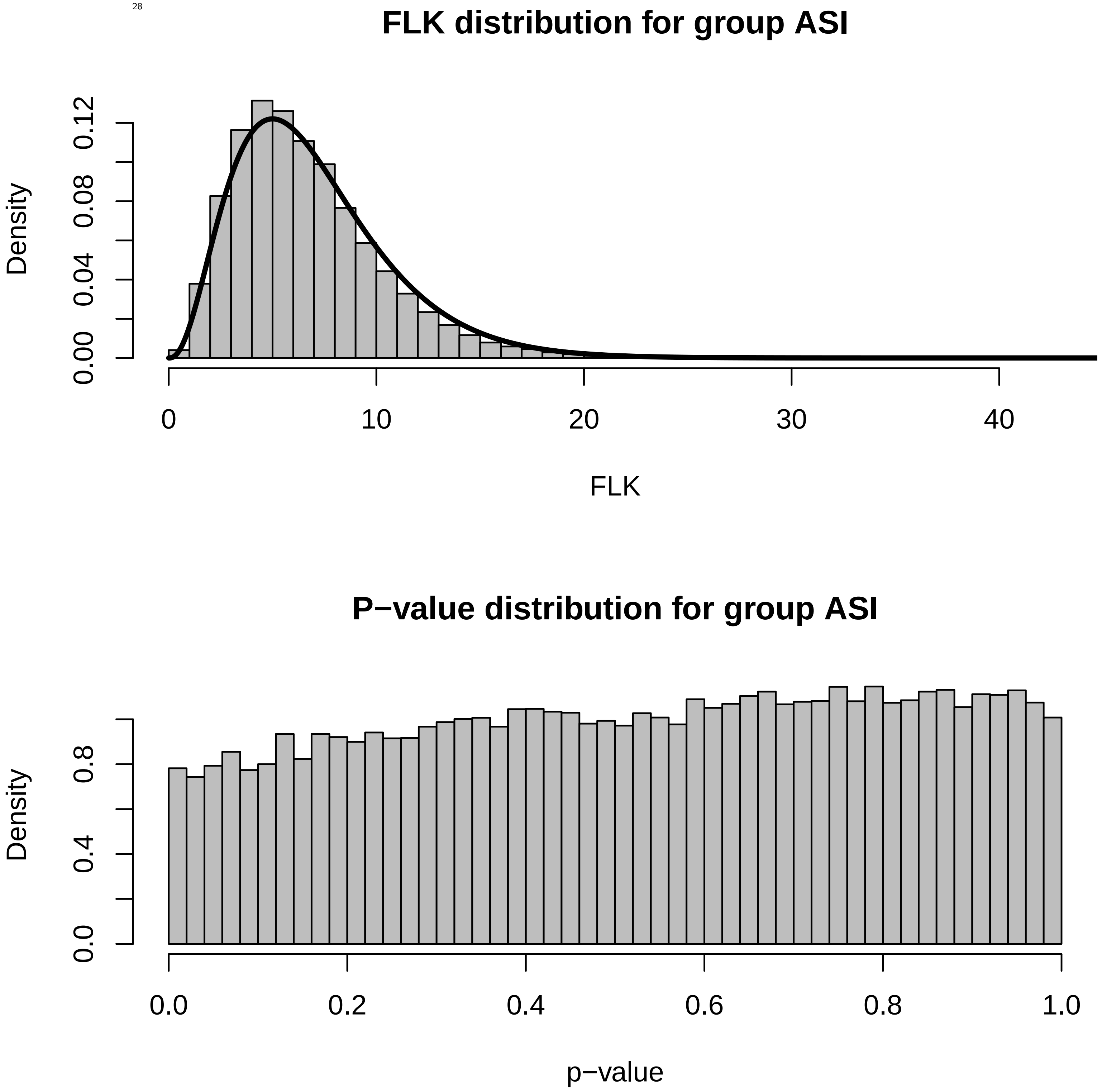
FLK distribution for group ASI
Density
0.00
0.04
0.08
0.12
0
10
20
30
40
FLK
P−value distribution for group ASI
Density
0.0
0.4
0.8
0.0
0.2
0.4
0.6
0.8
1.0
p−value

29

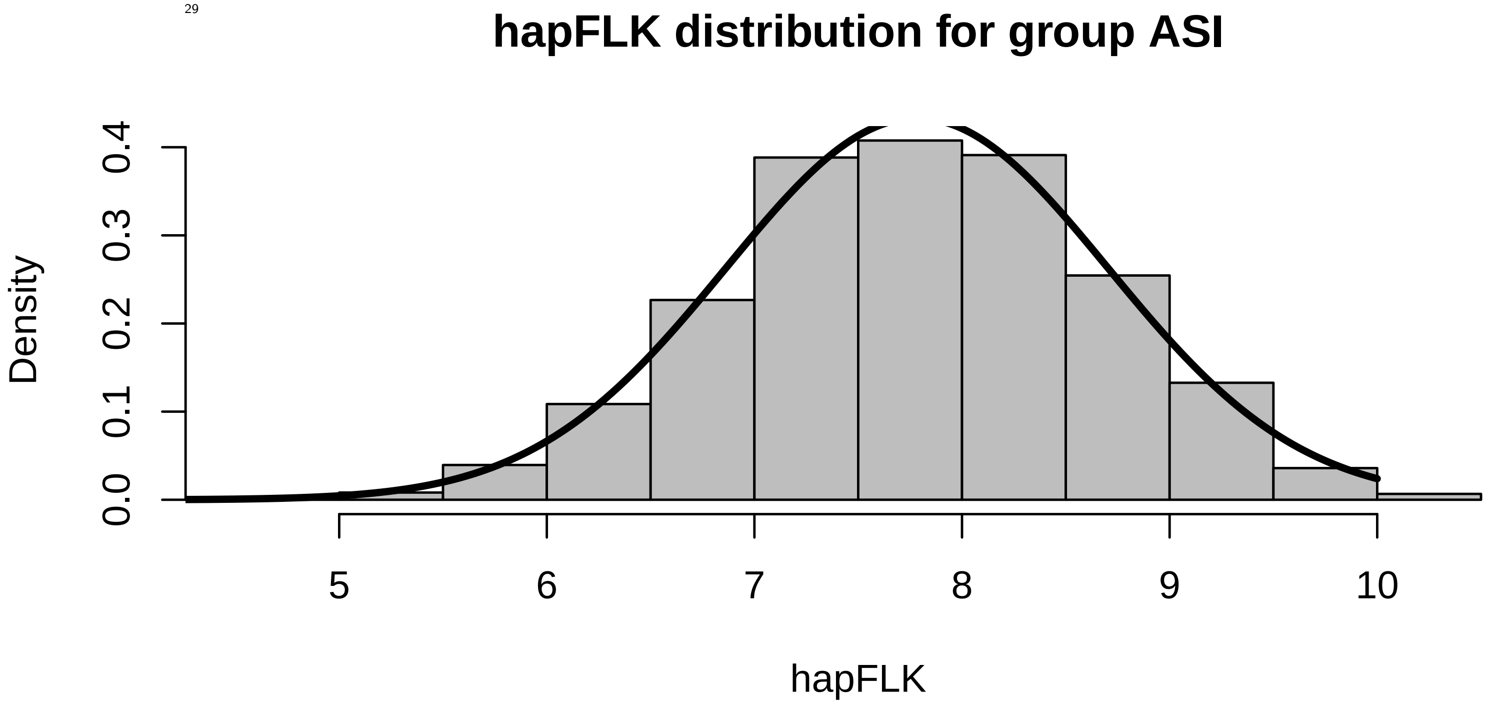
hapFLK distribution for group ASI
Density
0.0
0.1
0.2
0.3
0.4
5
6
7
8
9
10
hapFLK

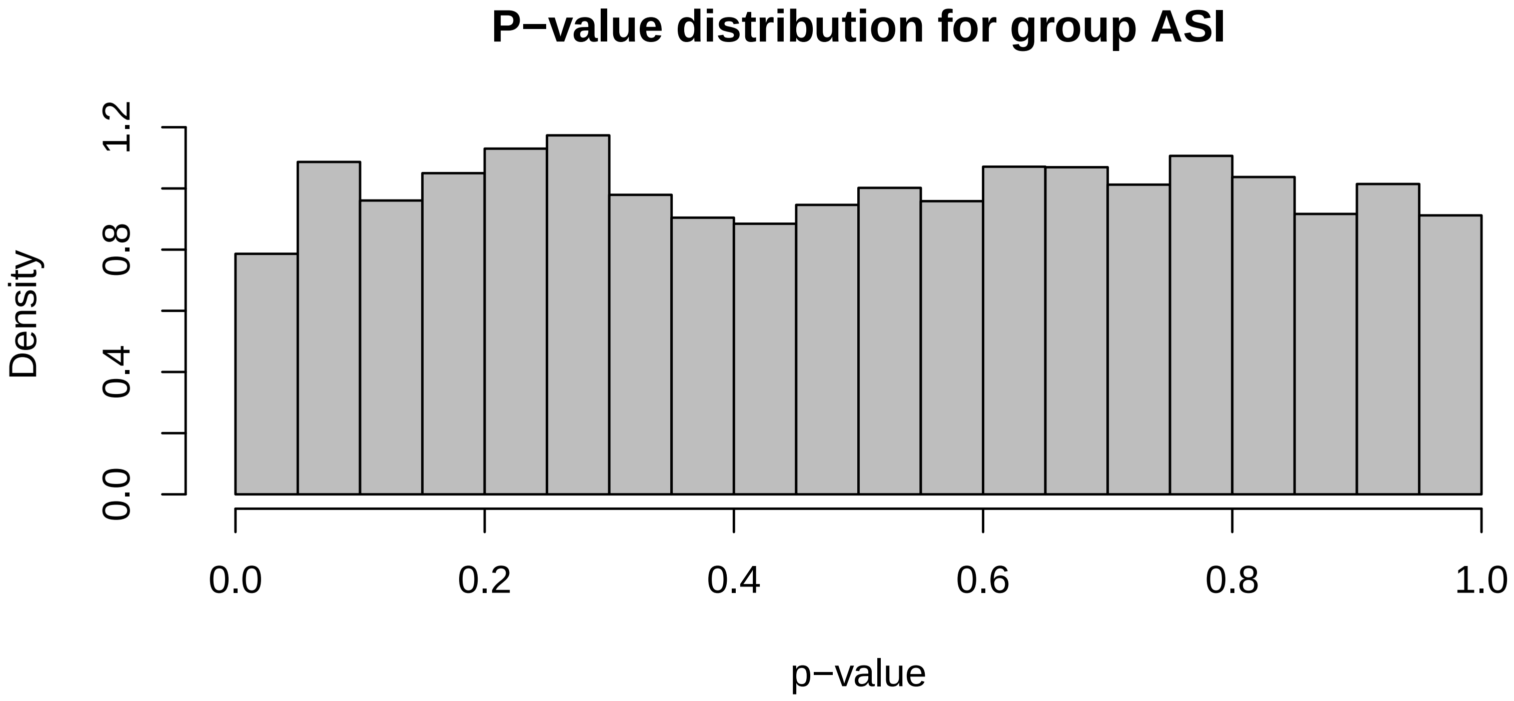
P−value distribution for group ASI
Density
0.0
0.4
0.8
1.2
0.0
0.2
0.4
0.6
0.8
1.0
p−value

FLK genome scan for group ASI

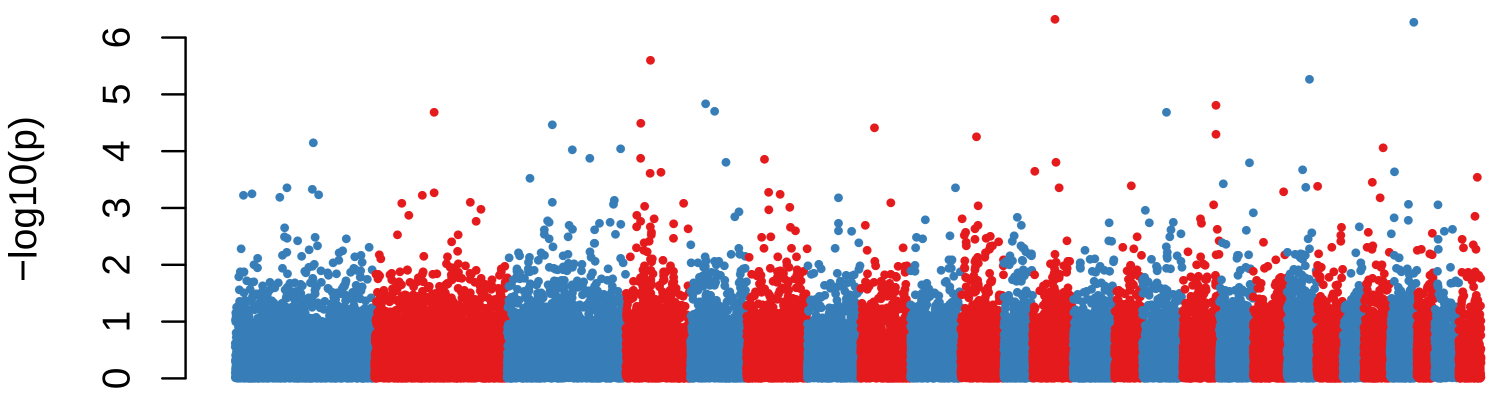

hapFLK genome scan for group ASI

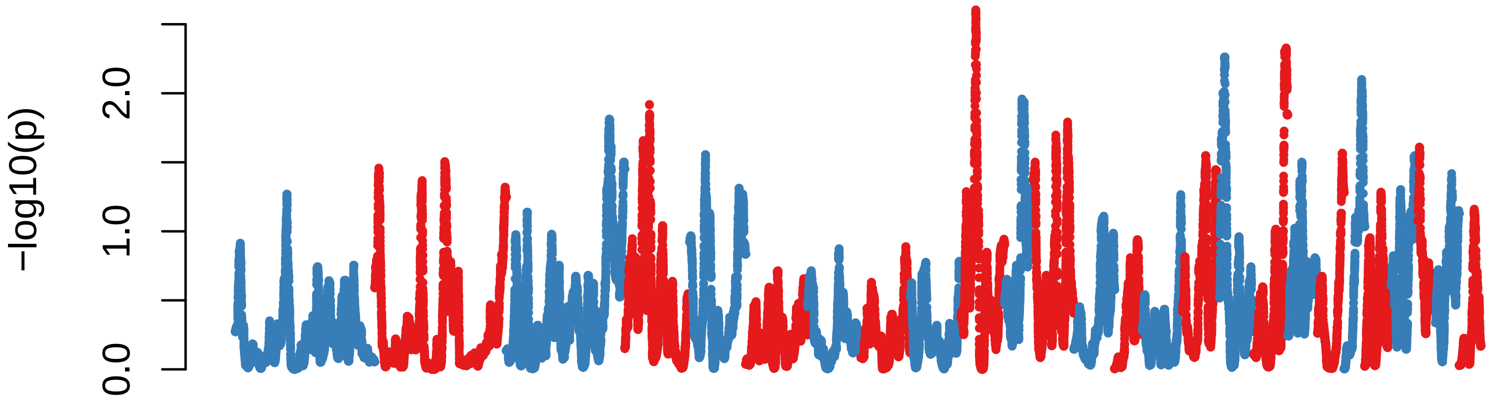

31

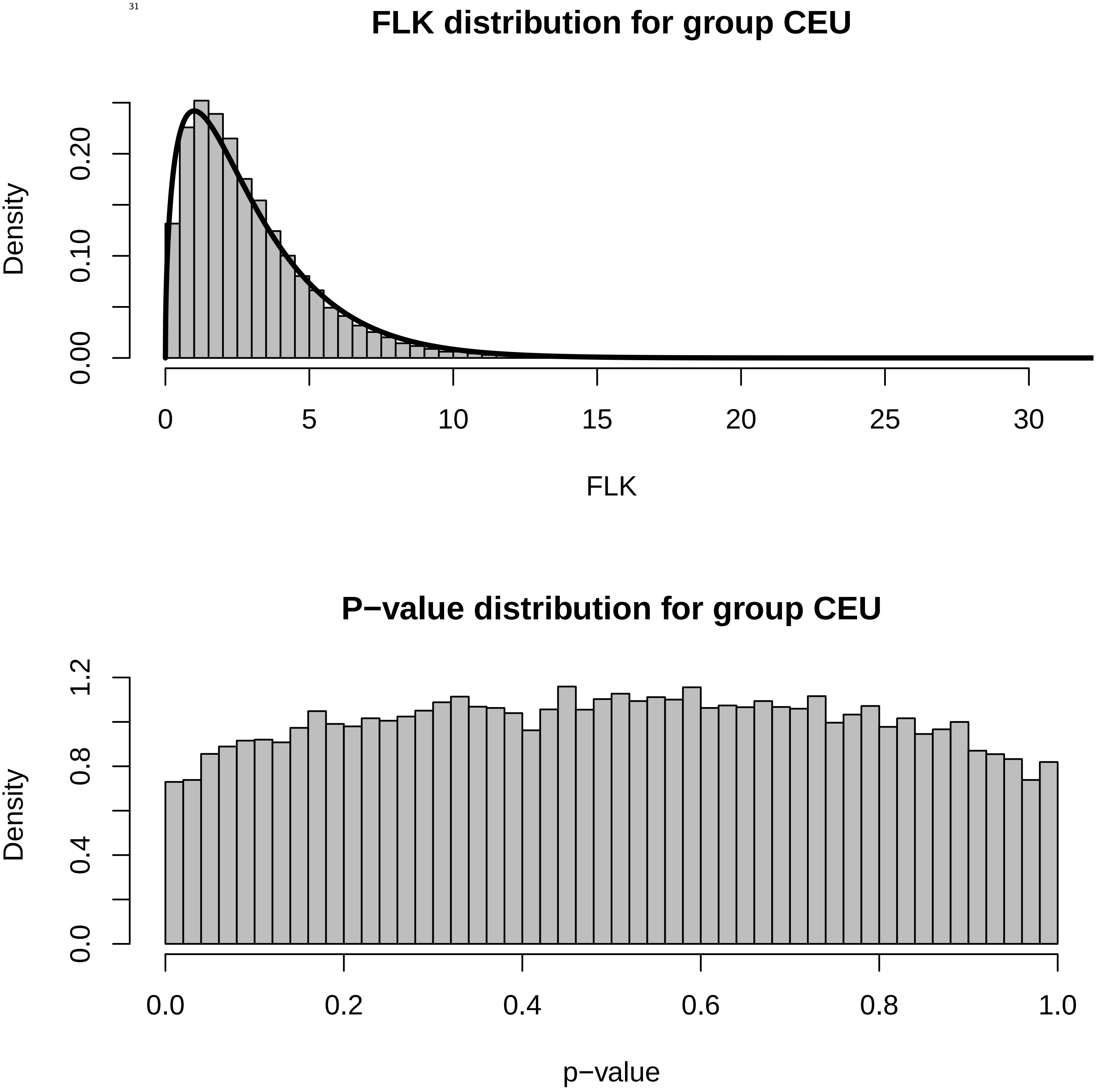
FLK distribution for group CEU
Density
0.00
0.10
0.20
0
5
10
15
20
25
30
FLK
P−value distribution for group CEU
Density
0.0
0.4
0.8
1.2
0.0
0.2
0.4
0.6
0.8
1.0
p−value

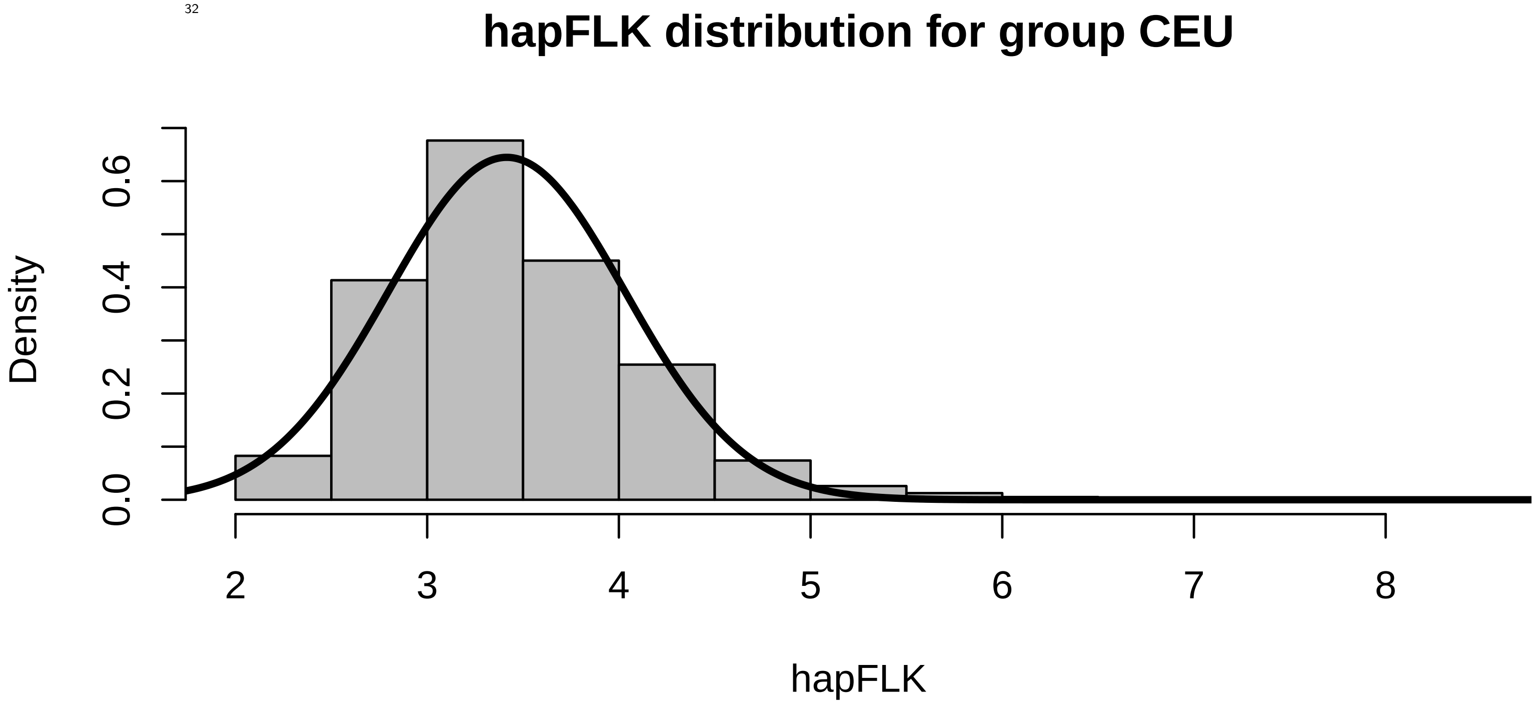
hapFLK distribution for group CEU
Density
0.0
0.2
0.4
0.6
2
3
4
5
6
7
8
hapFLK

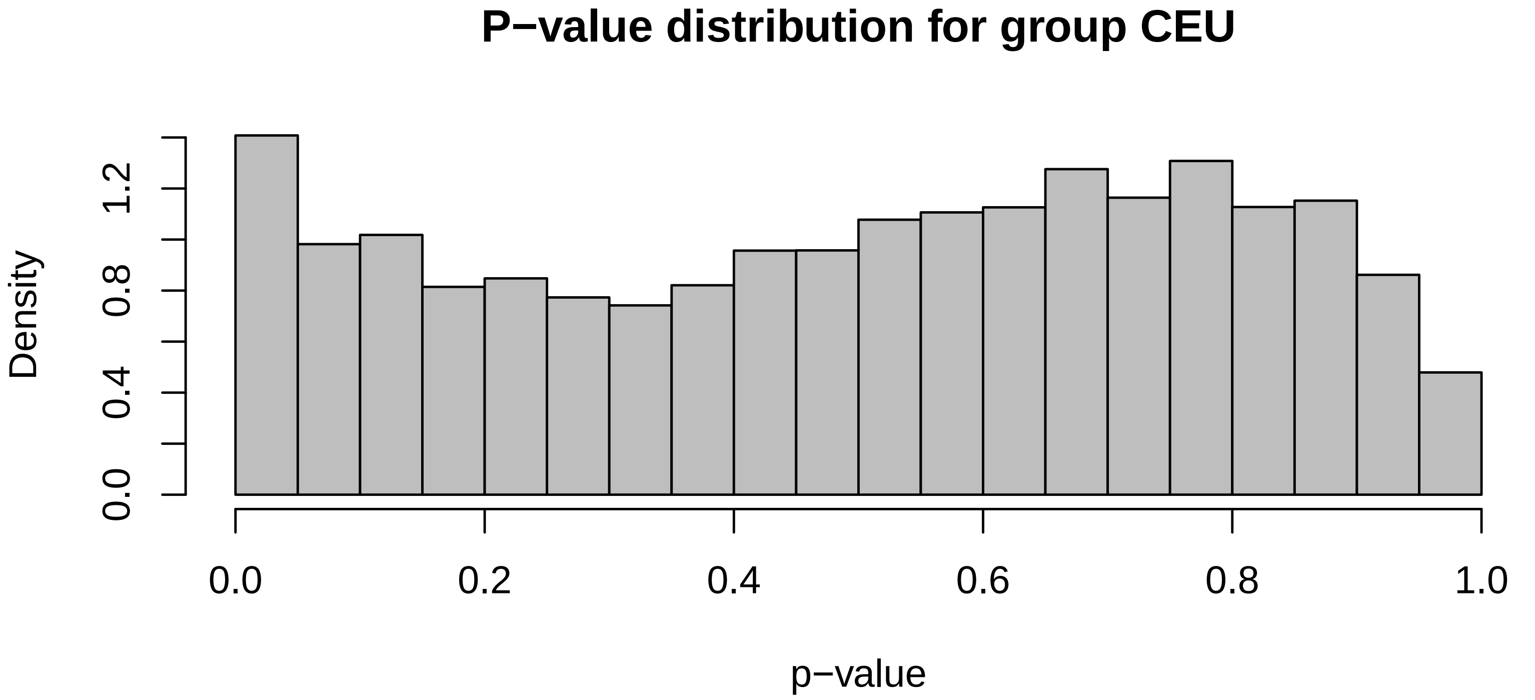
P−value distribution for group CEU
Density
0.0
0.4
0.8
1.2
0.0
0.2
0.4
0.6
0.8
1.0
p−value

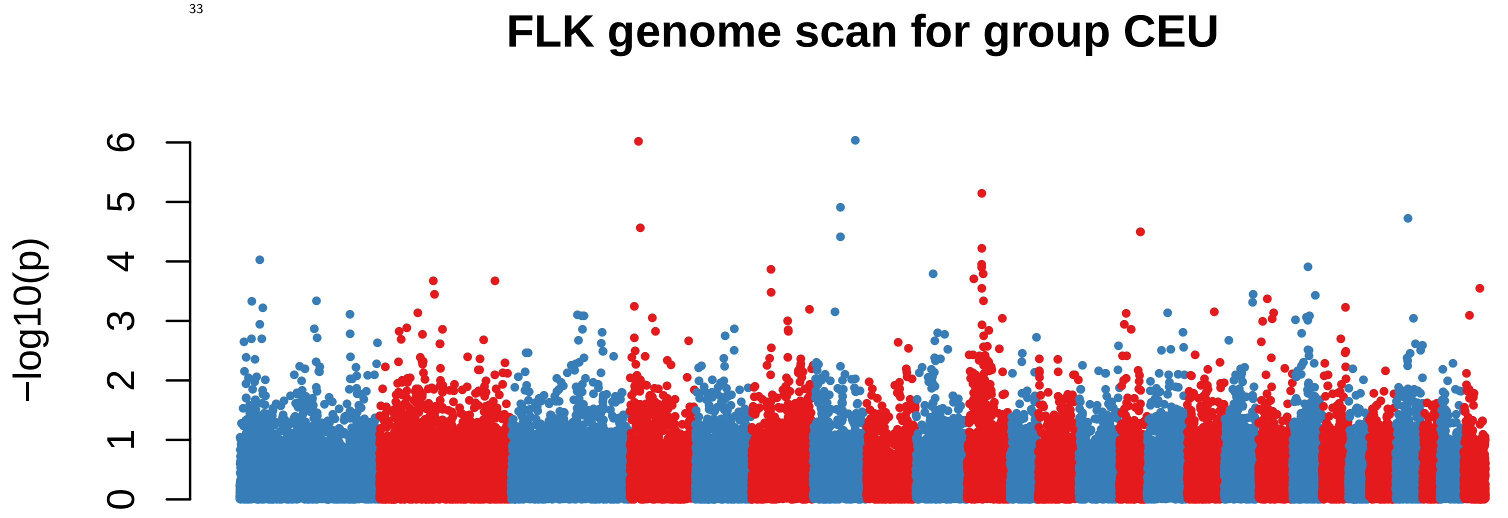
FLK genome scan for group CEU
−log10(p)
0
1
2
3
4
5
6

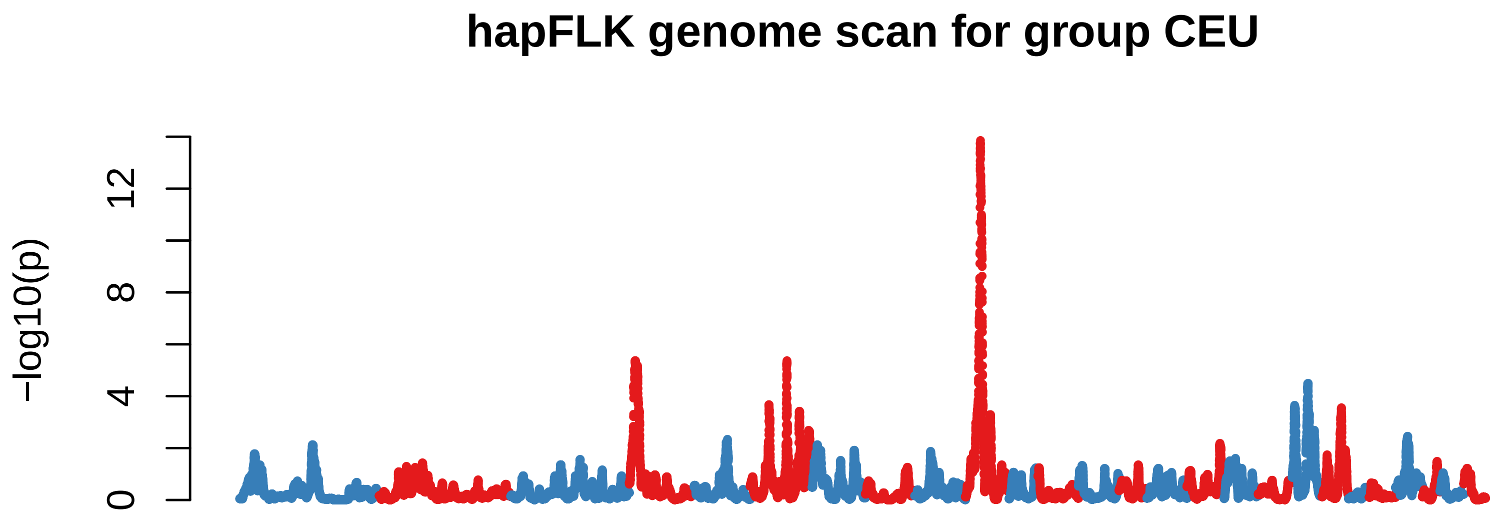
hapFLK genome scan for group CEU
−log10(p)
0
4
8
12

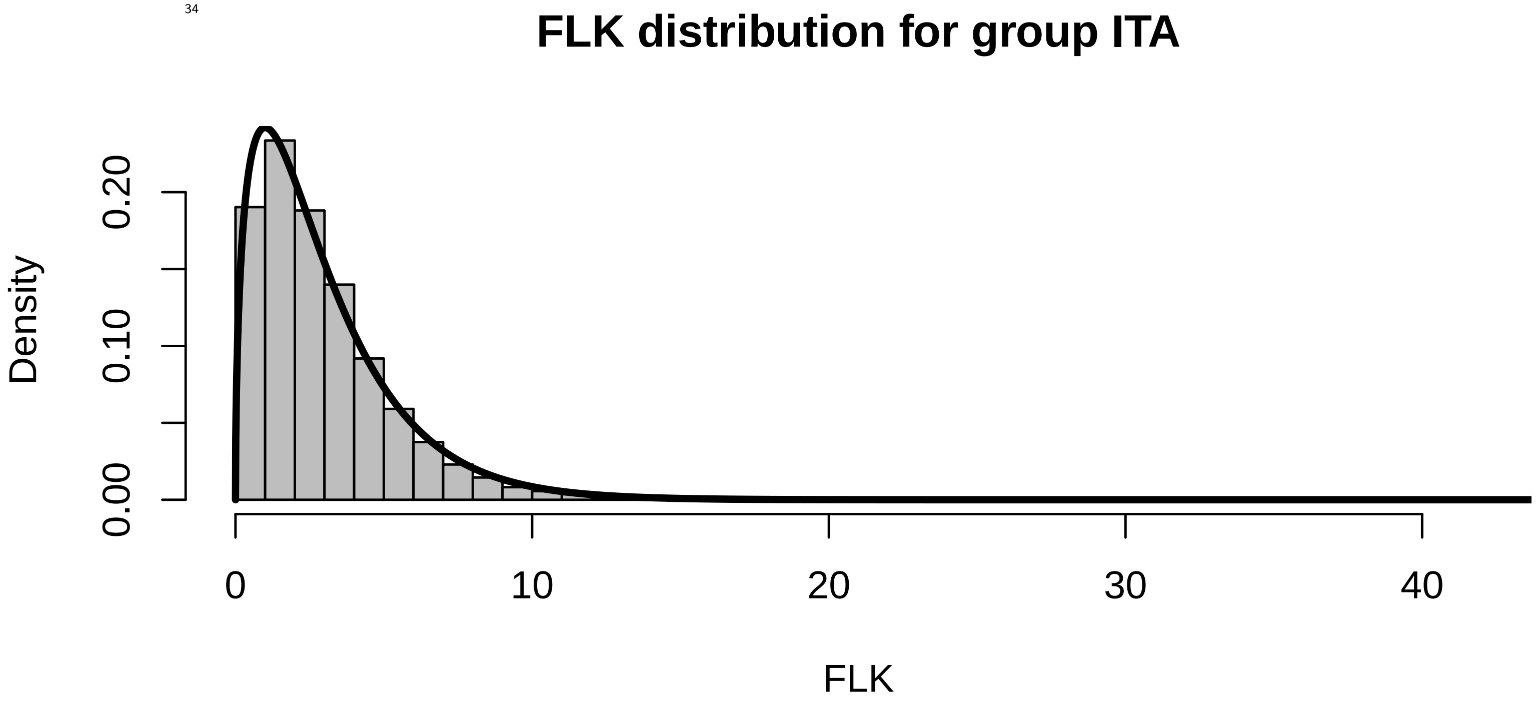
FLK distribution for group ITA
Density
0.00
0.10
0.20
0
10
20
30
40
FLK

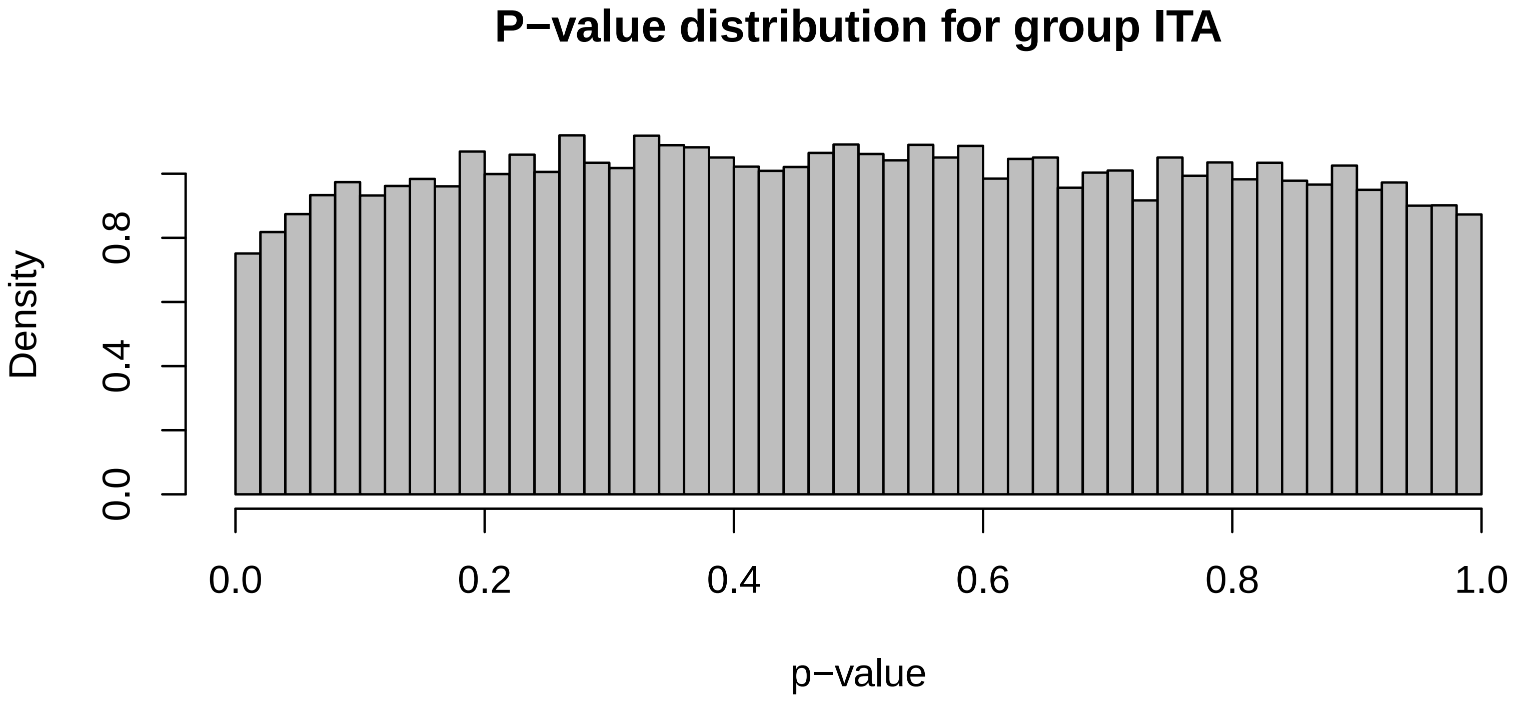
P−value distribution for group ITA
Density
0.0
0.4
0.8
0.0
0.2
0.4
0.6
0.8
1.0
p−value

35

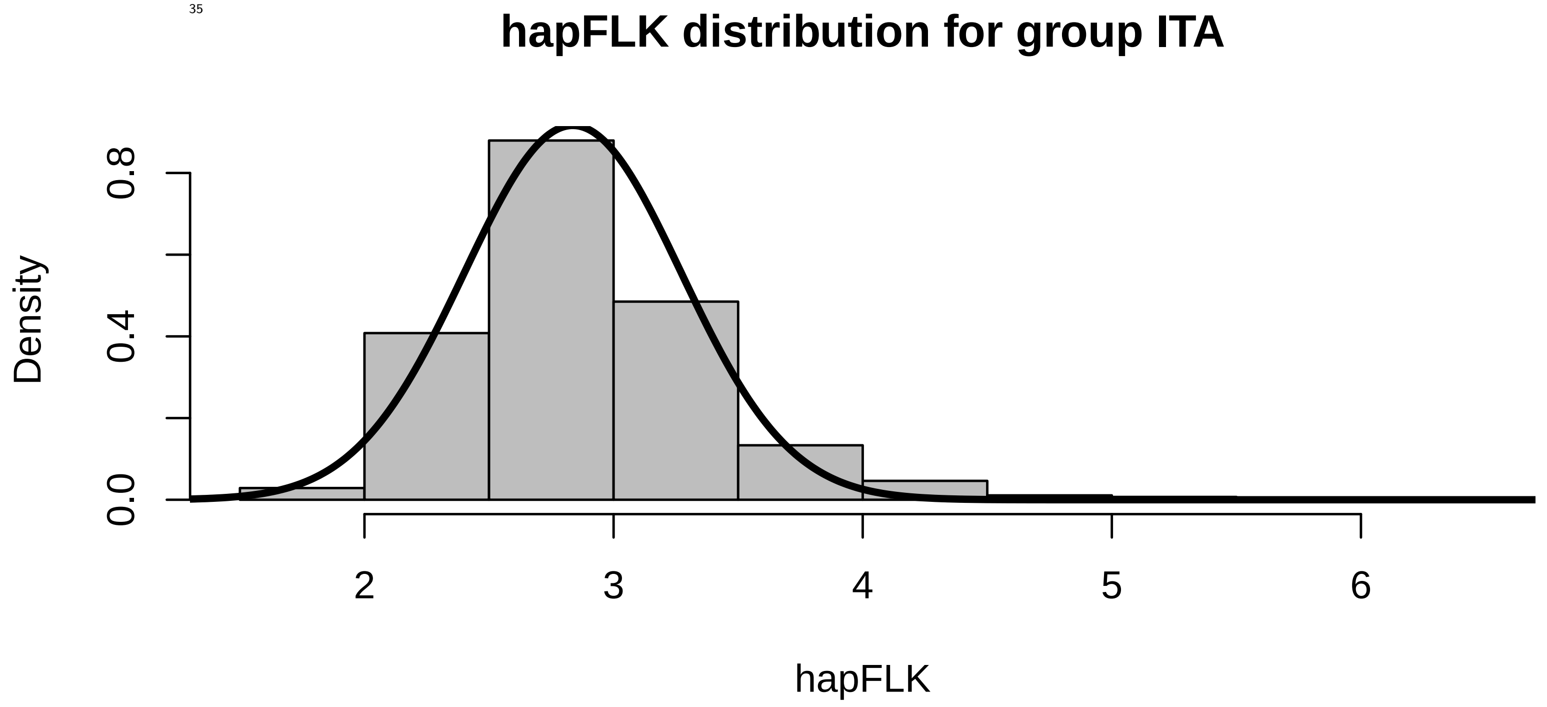
hapFLK distribution for group ITA
Density
0.0
0.4
0.8
2
3
4
5
6
hapFLK

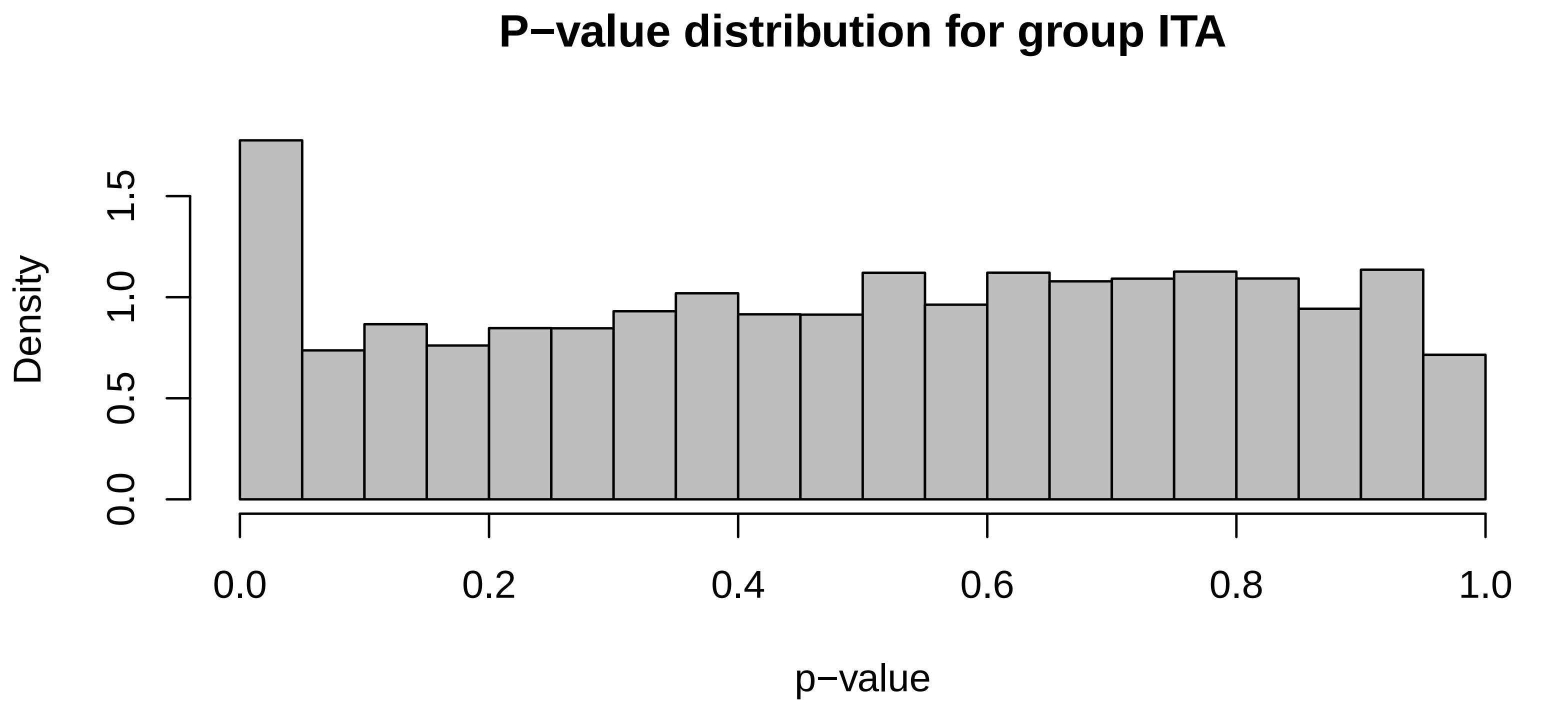
P−value distribution for group ITA
Density
0.0
0.5
1.0
1.5
0.0
0.2
0.4
0.6
0.8
1.0
p−value

36

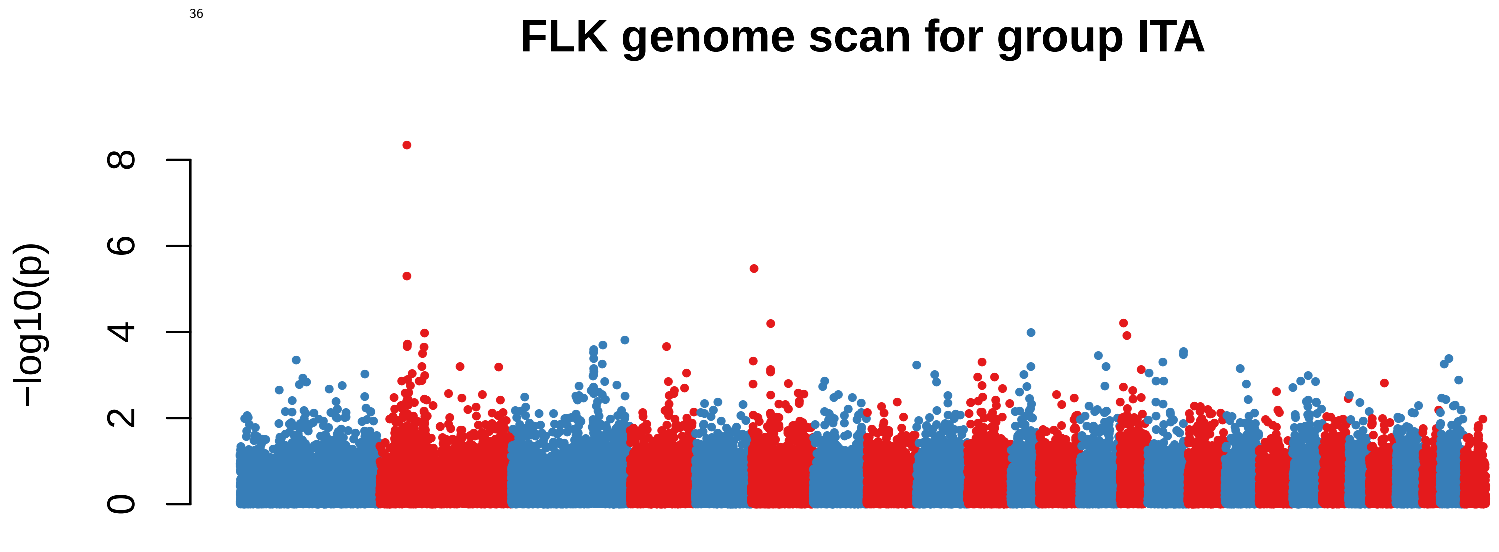
FLK genome scan for group ITA
−log10(p)
0
2
4
6
8

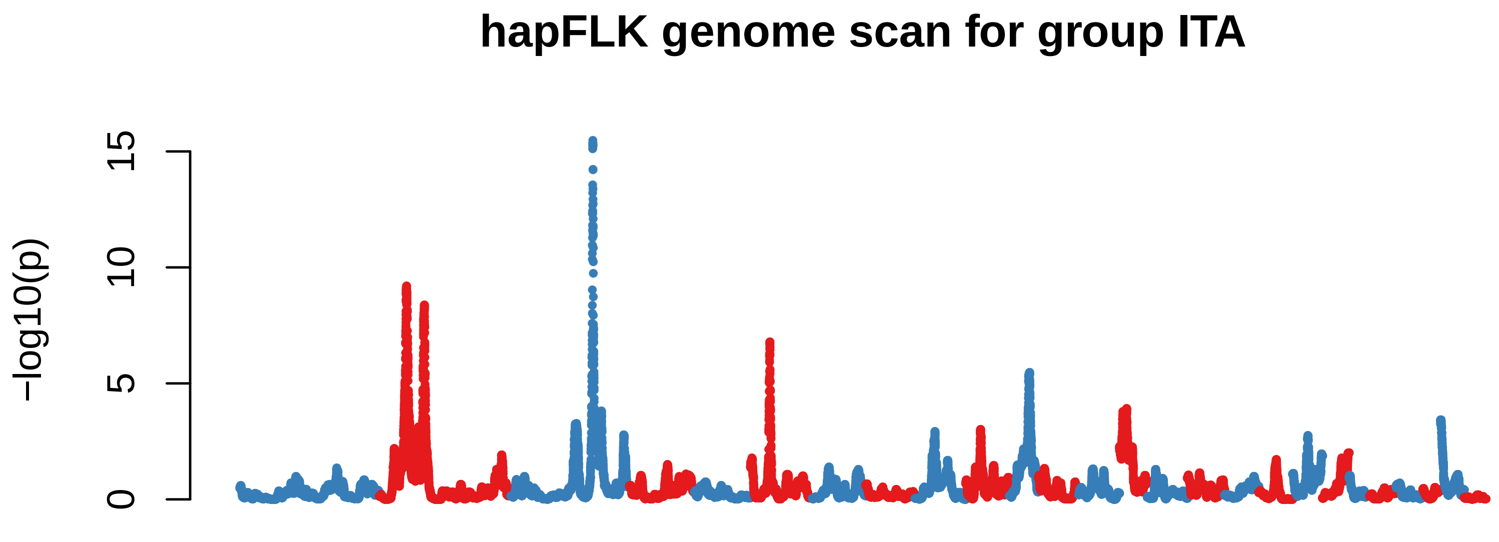
hapFLK genome scan for group ITA
−log10(p)
0
5
10
15

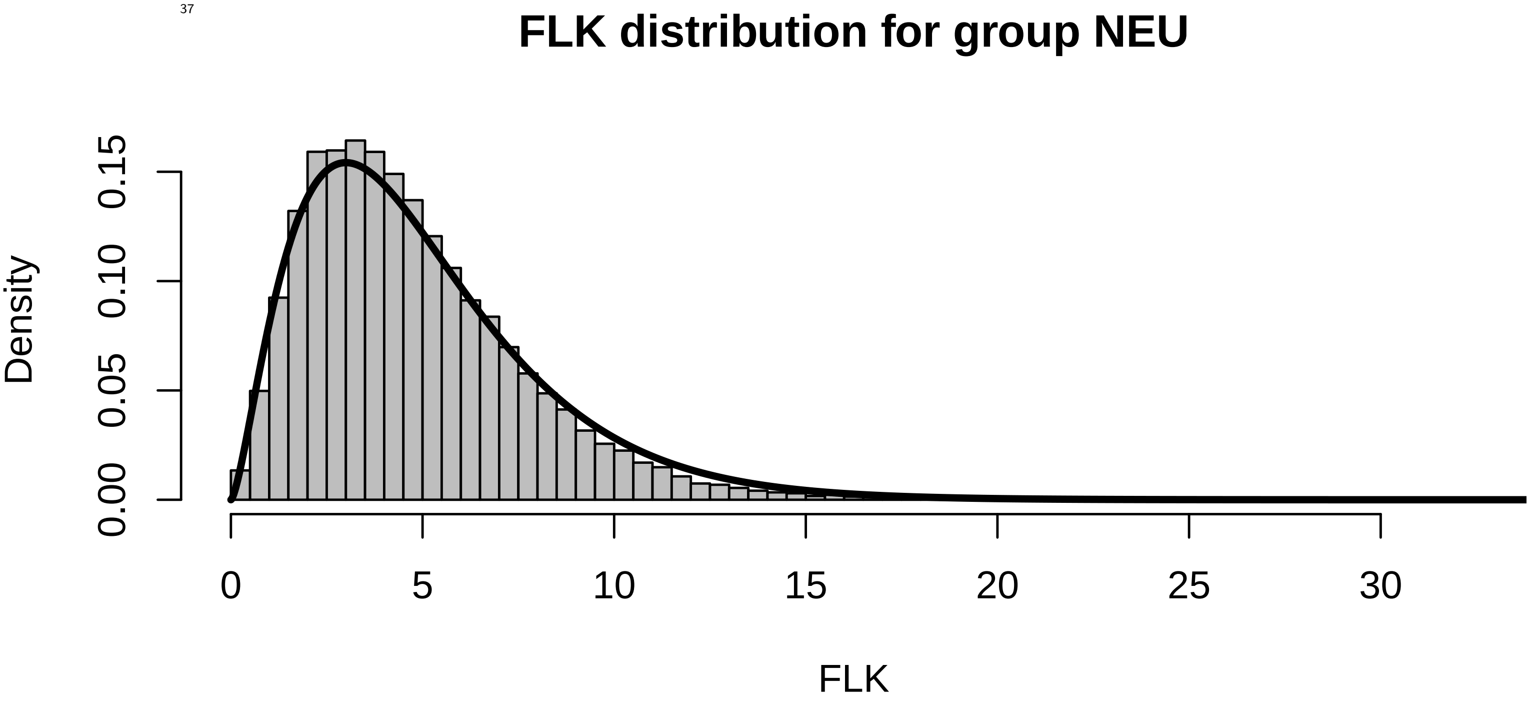
FLK distribution for group NEU
Density
0.00
0.05
0.10
0.15
0
5
10
15
20
25
30
FLK

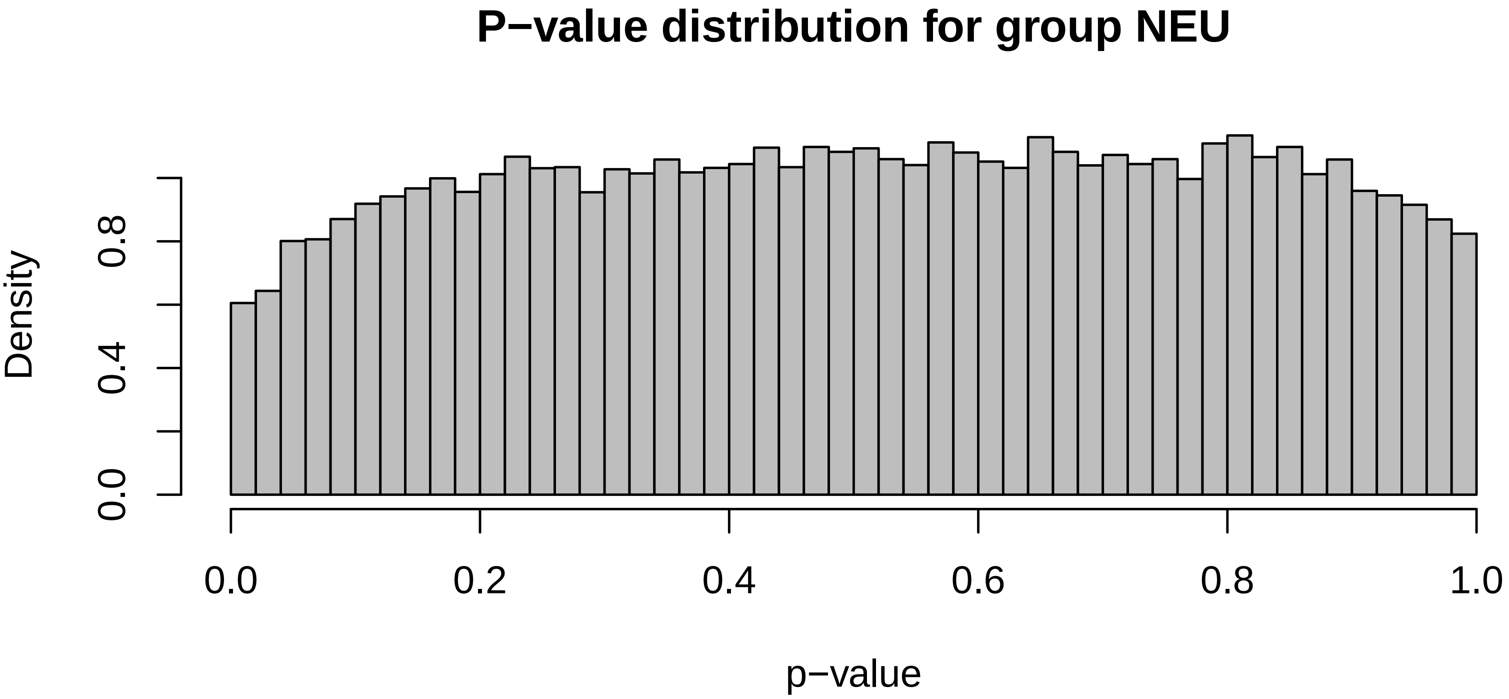
P−value distribution for group NEU
Density
0.0
0.4
0.8
0.0
0.2
0.4
0.6
0.8
1.0
p−value

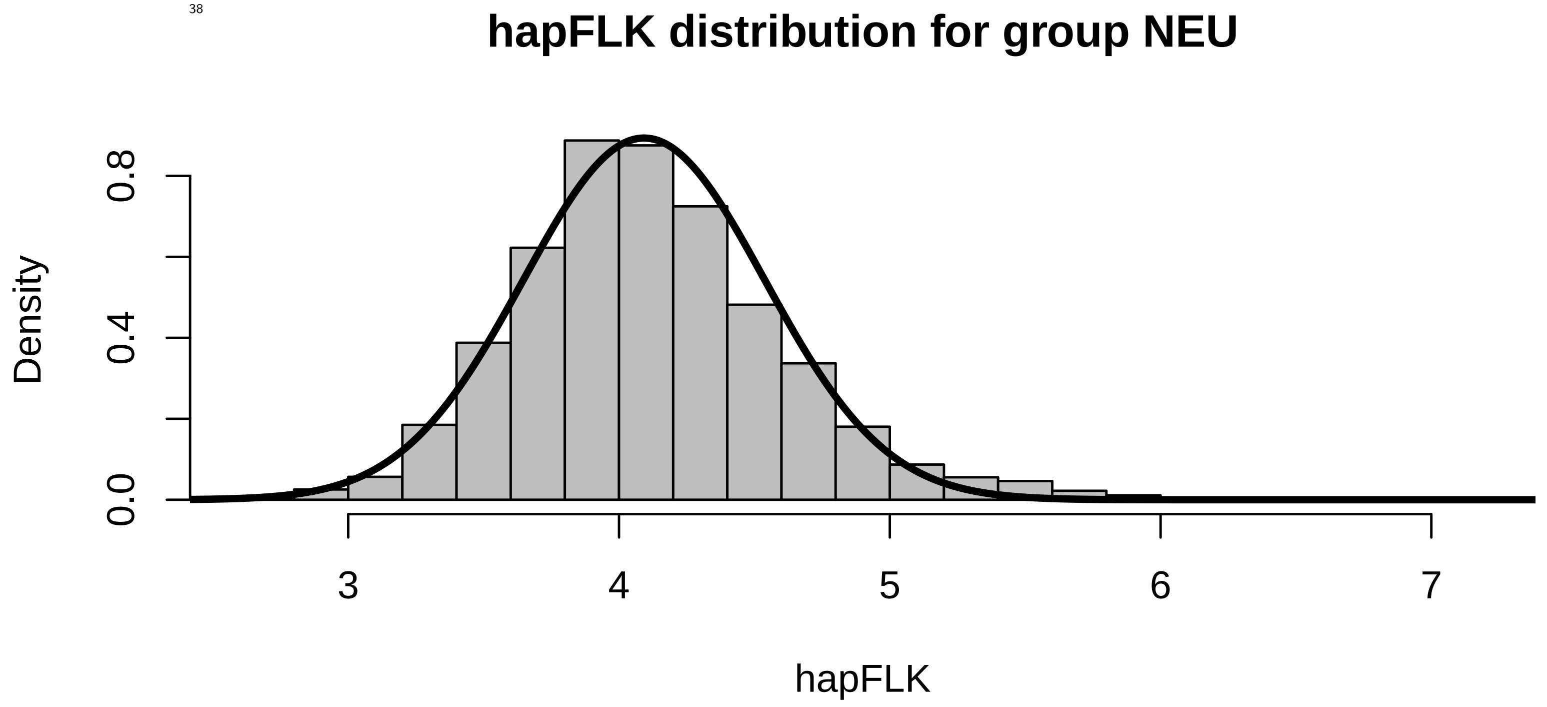
hapFLK distribution for group NEU
Density
0.0
0.4
0.8
3
4
5
6
7
hapFLK

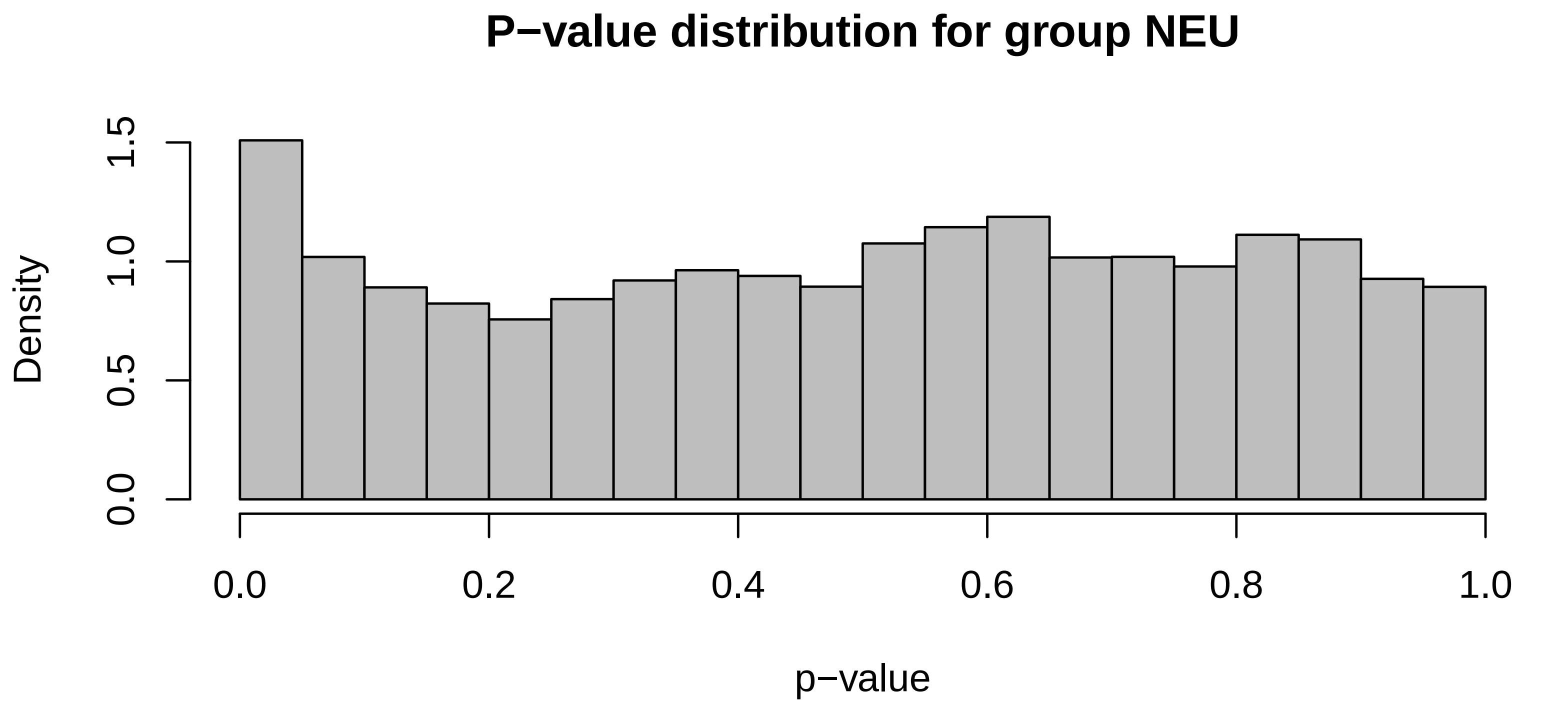
P−value distribution for group NEU
Density
0.0
0.5
1.0
1.5
0.0
0.2
0.4
0.6
0.8
1.0
p−value

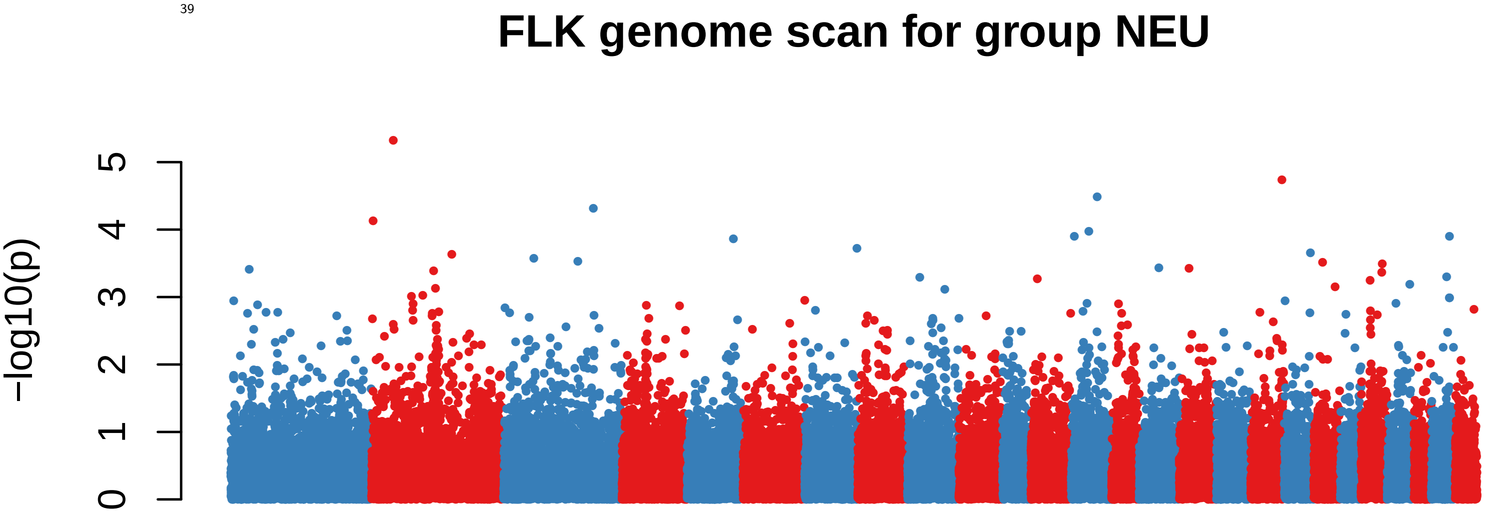
FLK genome scan for group NEU
−log10(p)
0
1
2
3
4
5

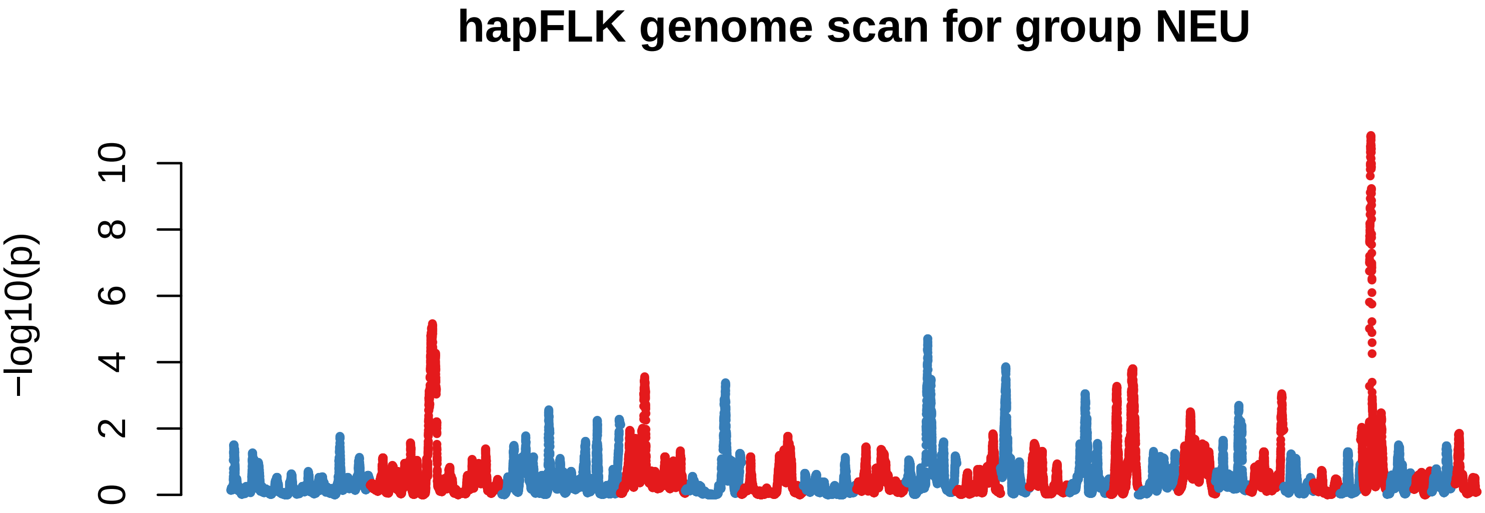
hapFLK genome scan for group NEU
−log10(p)
0
2
4
6
8
10

FLK distribution for group SWA

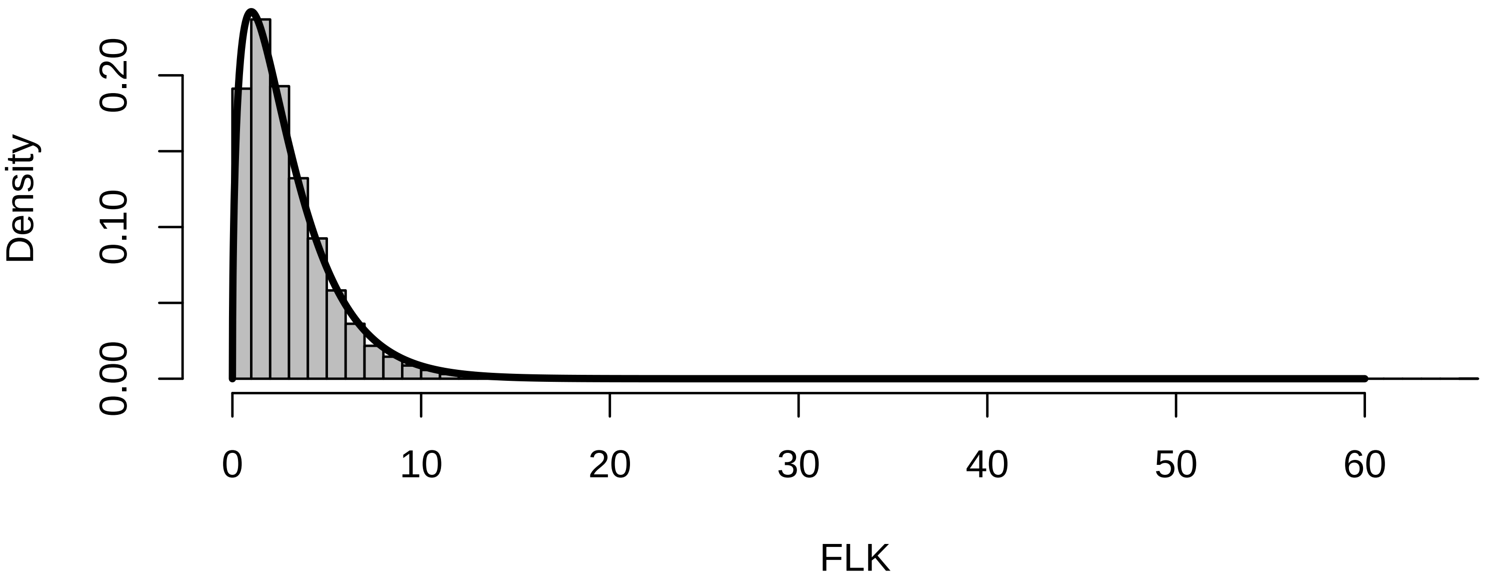

P−value distribution for group SWA

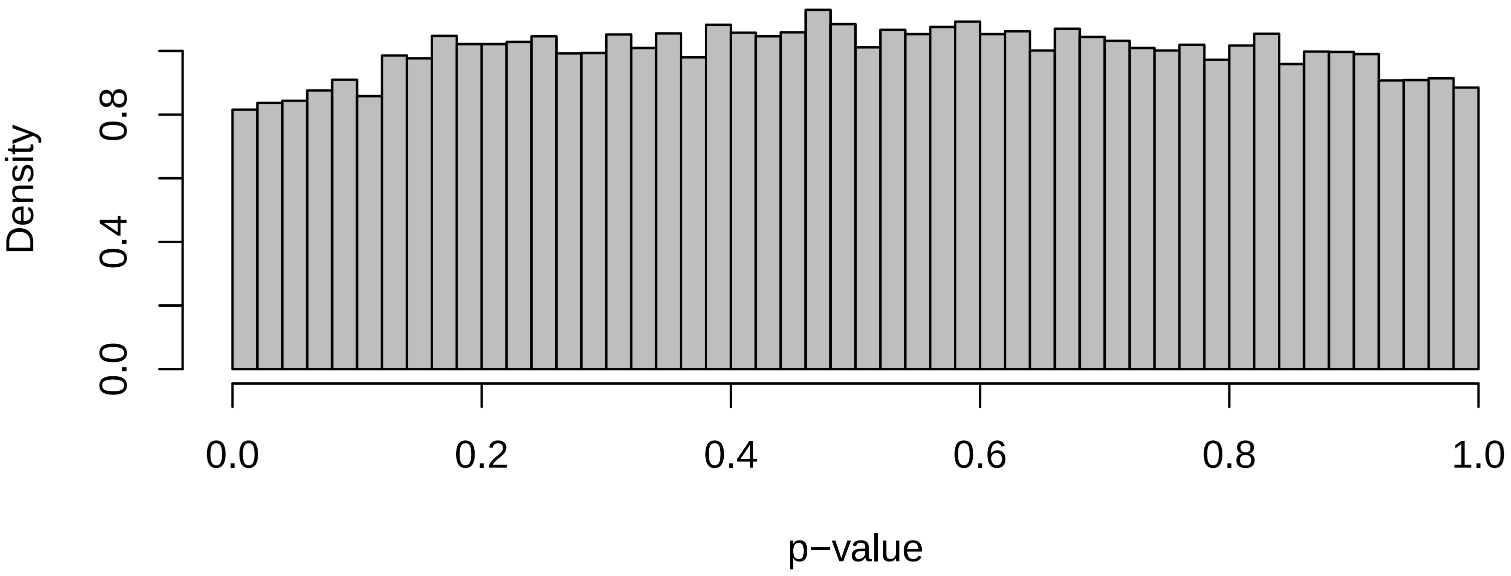

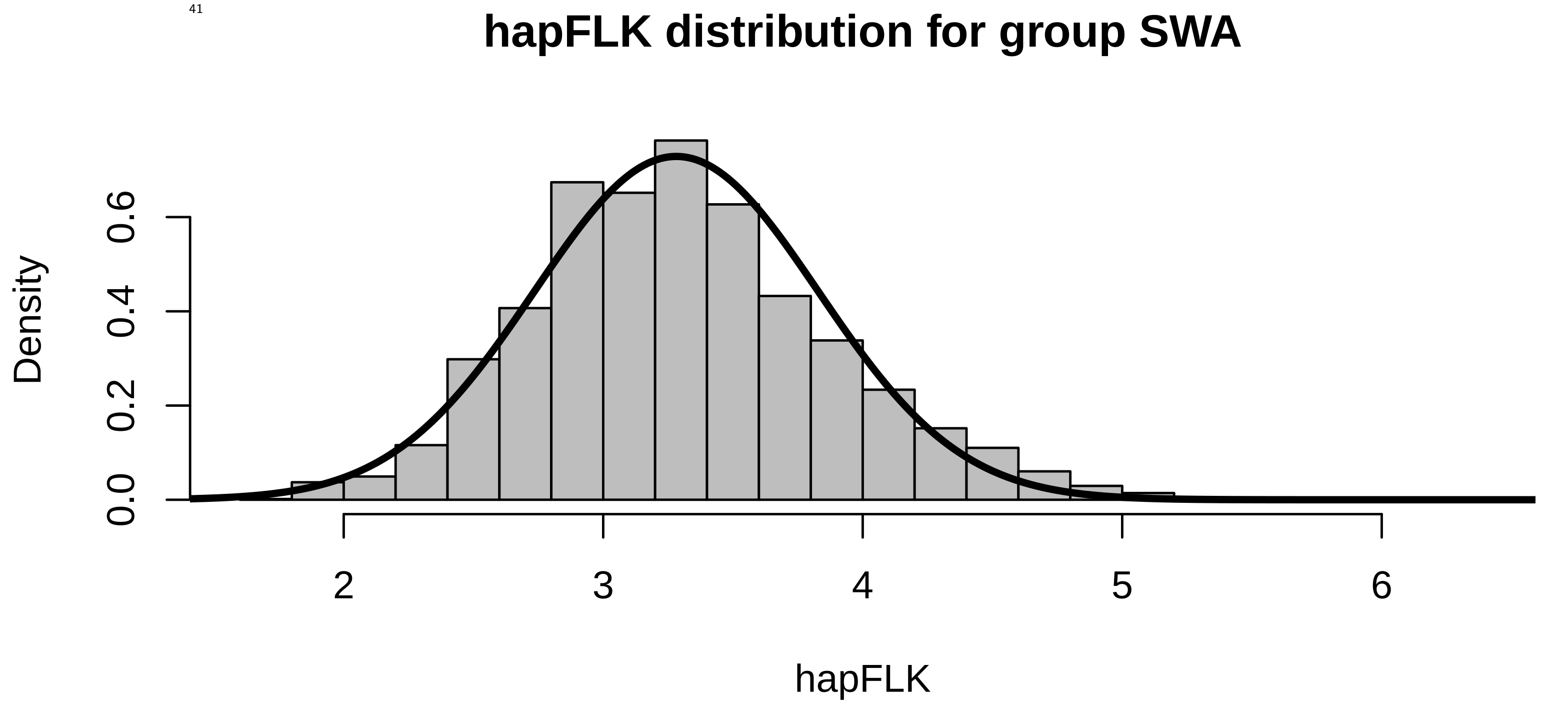
hapFLK distribution for group SWA
Density
0.0
0.2
0.4
0.6
2
3
4
5
6
hapFLK

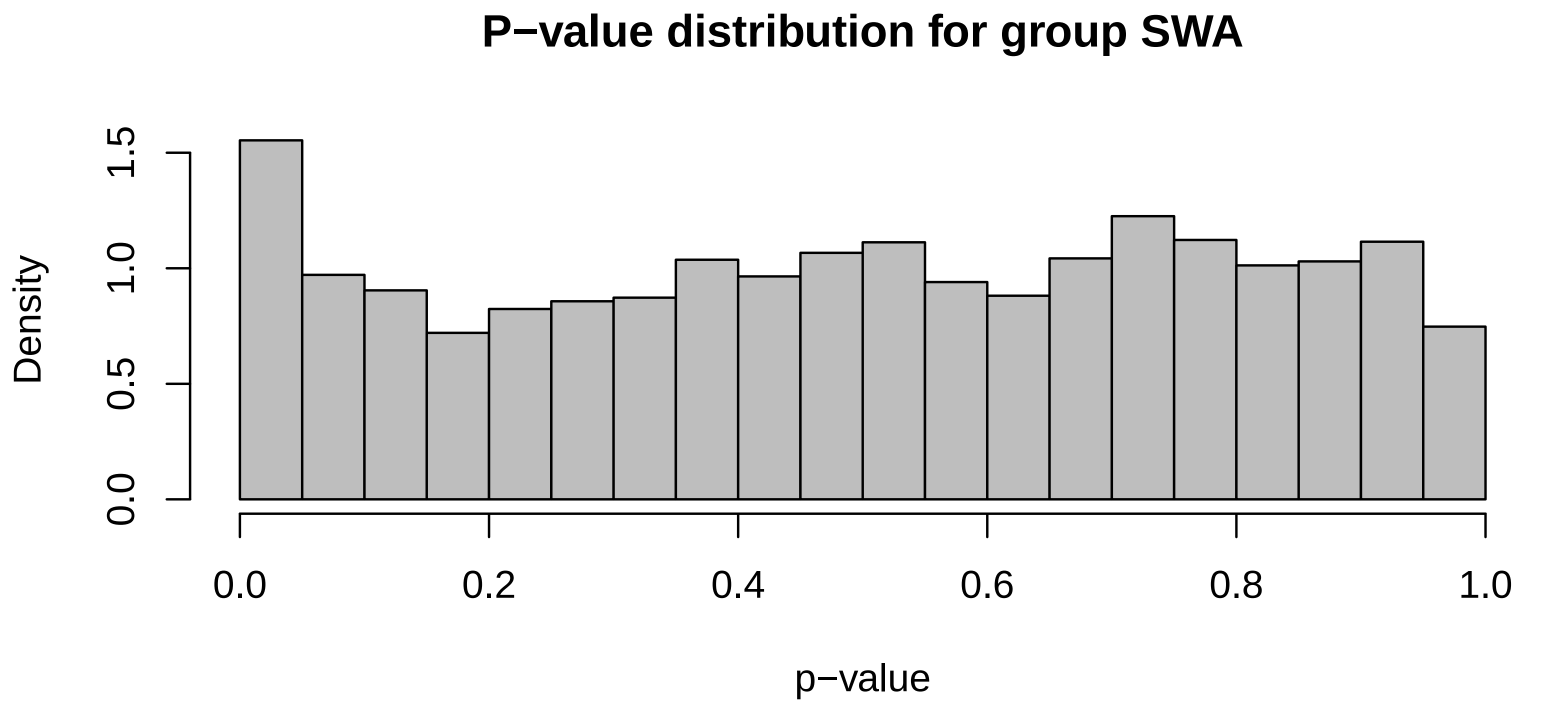
P−value distribution for group SWA
Density
0.0
0.5
1.0
1.5
0.0
0.2
0.4
0.6
0.8
1.0
p−value

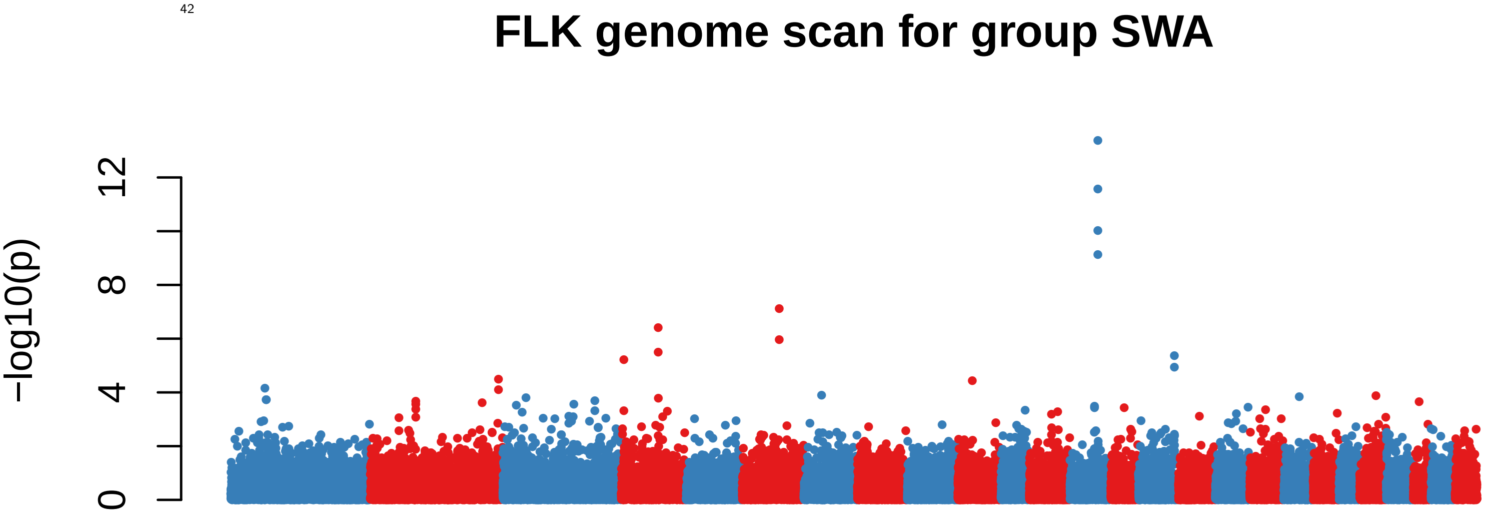
FLK genome scan for group SWA
−log10(p)
0
4
8
12

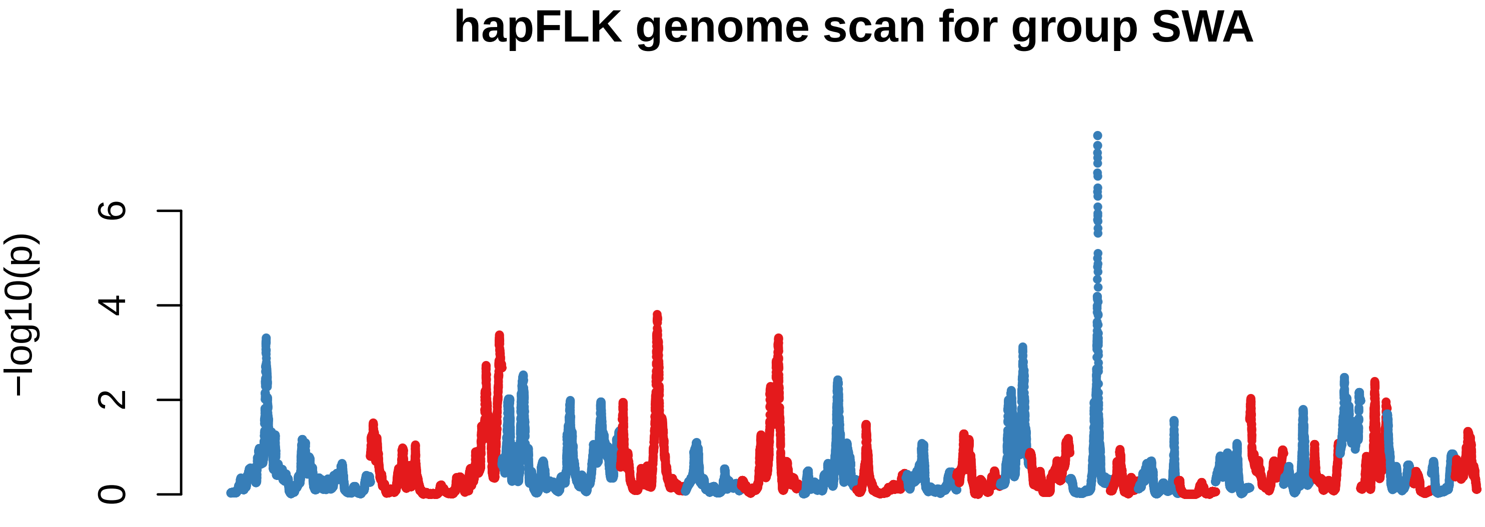
hapFLK genome scan for group SWA
−log10(p)
0
2
4
6

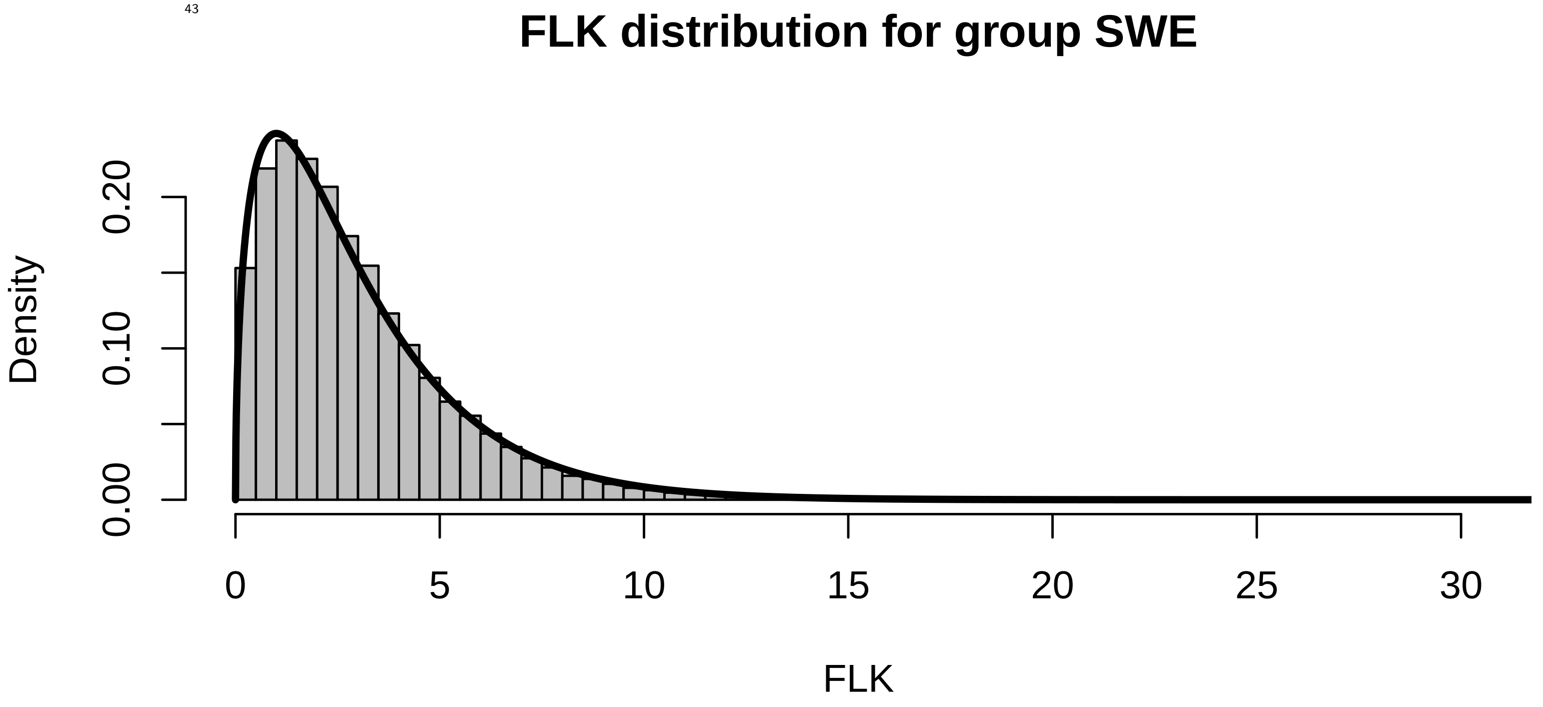
FLK distribution for group SWE
Density
0.00
0.10
0.20
0
5
10
15
20
25
30
FLK

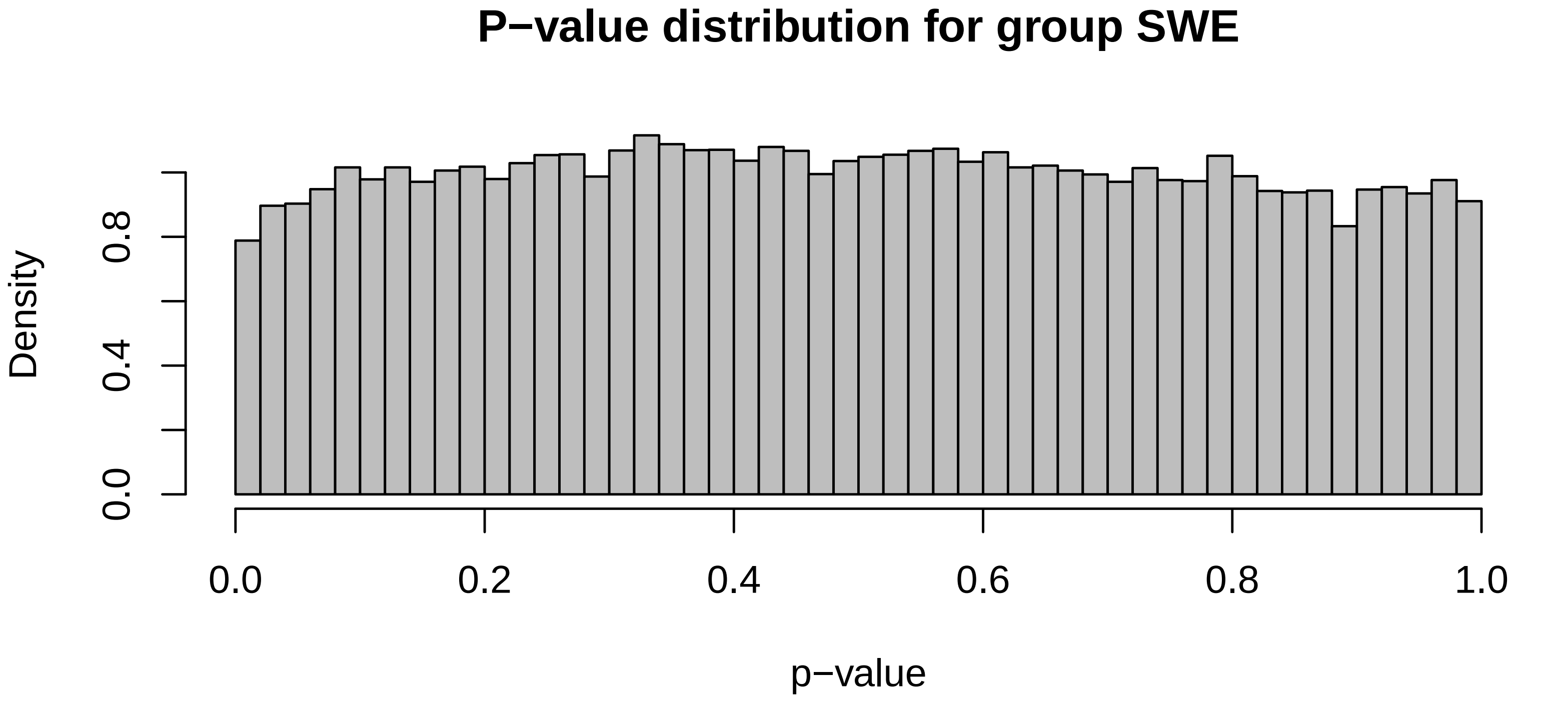
P−value distribution for group SWE
Density
0.0
0.4
0.8
0.0
0.2
0.4
0.6
0.8
1.0
p−value

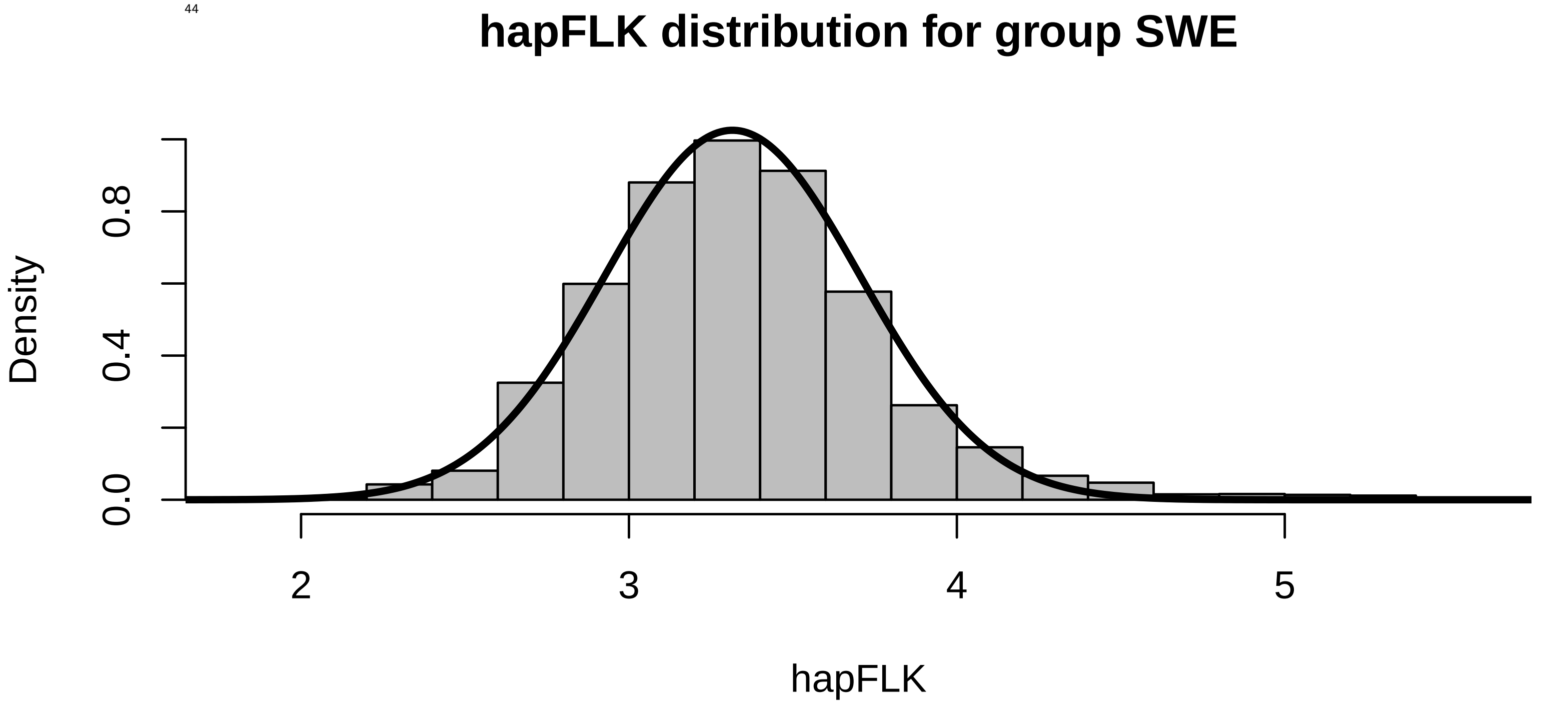
hapFLK distribution for group SWE
Density
0.0
0.4
0.8
2
3
4
5
hapFLK

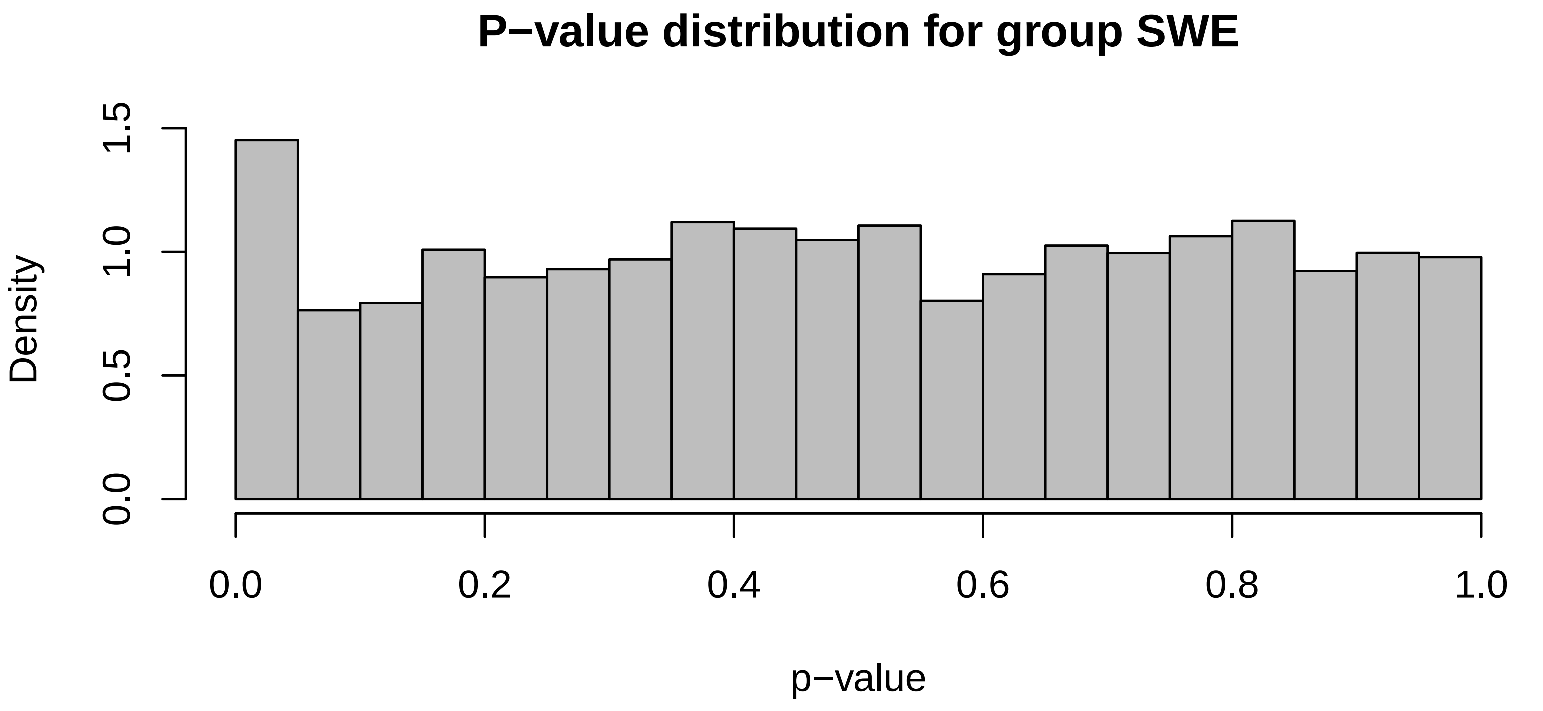
P−value distribution for group SWE
Density
0.0
0.5
1.0
1.5
0.0
0.2
0.4
0.6
0.8
1.0
p−value

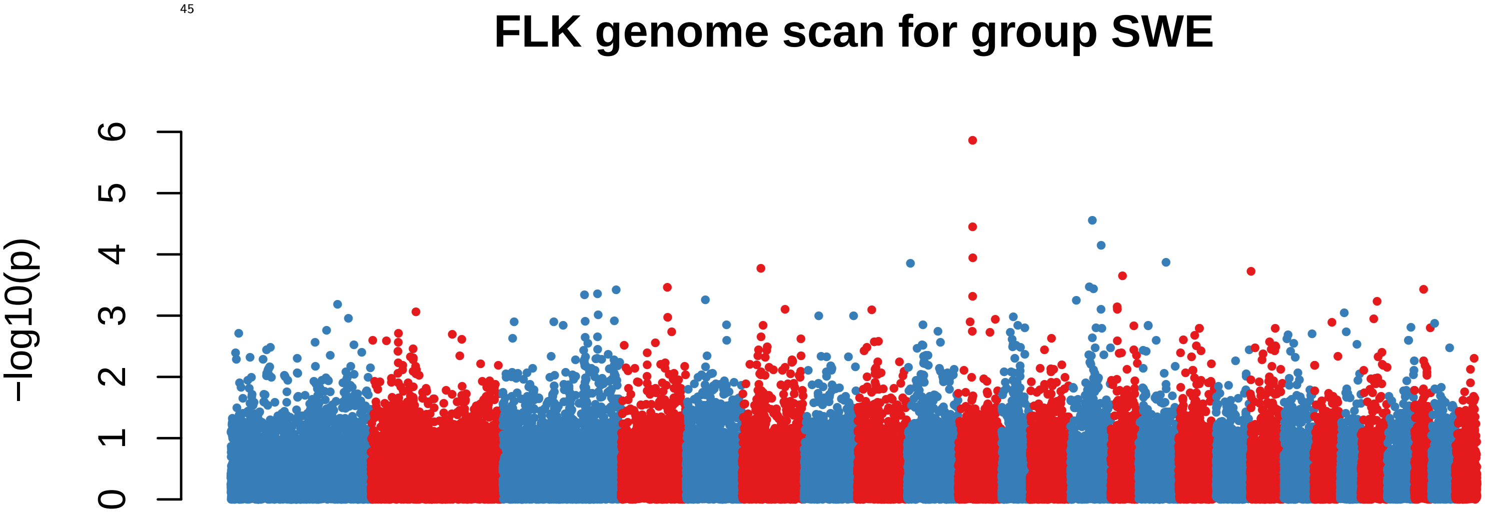
FLK genome scan for group SWE
−log10(p)
0
1
2
3
4
5
6

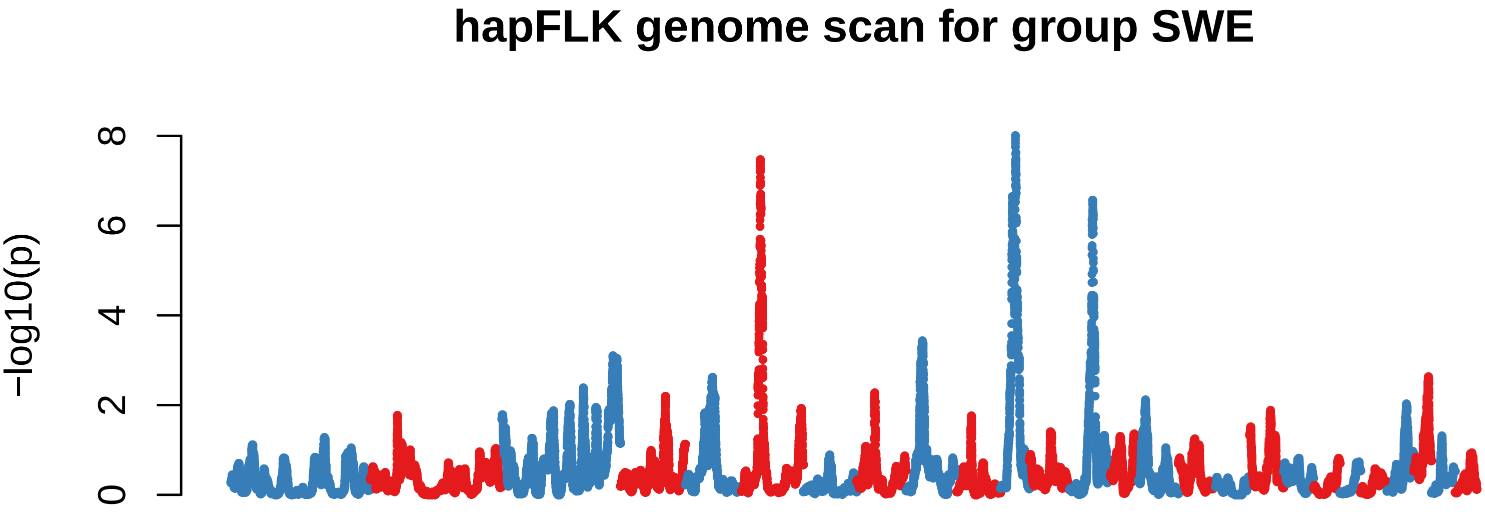
hapFLK genome scan for group SWE
−log10(p)
0
2
4
6
8
